# *Bacillus velezensis* 83 a bacterial strain from mango phyllosphere, useful for biological control and plant growth promotion

**DOI:** 10.1186/s13568-020-01101-8

**Published:** 2020-09-07

**Authors:** Karina A. Balderas-Ruíz, Patricia Bustos, Rosa I. Santamaria, Víctor González, Sergio Andrés Cristiano-Fajardo, Salvador Barrera-Ortíz, Miriam Mezo-Villalobos, Sergio Aranda-Ocampo, Ángel Arturo Guevara-García, Enrique Galindo, Leobardo Serrano-Carreón

**Affiliations:** 1 Departamento de Ingeniería Celular y Biocatálisis, Instituto de Biotecnología, Universidad Nacional Autónoma de México, Av. Universidad #2001, Col. Chamilpa, C. P. 62210 Cuernavaca, Morelos México; 2 Centro de Ciencias Genómicas, Universidad Nacional Autónoma de México, Av. Universidad #2001, Col. Chamilpa, C. P. 62210 Cuernavaca, Morelos México; 3 Departamento de Biología Molecular de Plantas, Instituto de Biotecnología, Universidad Nacional Autónoma de México, Av. Universidad #2001, Col. Chamilpa, C. P. 62210 Cuernavaca, Morelos México; 4 Agro&Biotecnia S de RL de CV, Limones #8, Col. Amate Redondo, C. P. 62334 Cuernavaca, Morelos México; 5 Departamento Fitosanidad-Fitopatología, Colegio de Postgraduados, Carretera México-Texcoco Km. 36.5 Montecillo, Texcoco, C.P. 56230 Edo. de México México

**Keywords:** *Bacillus velezensis* 83, Genome sequencing, Biological control agent, PGPB, Secondary metabolites production

## Abstract

*Bacillus velezensis* 83 was isolated from mango tree phyllosphere of orchards located in El Rosario, Sinaloa, México. The assessment of this strain as BCA (biological control agent), as well as PGPB (plant growth-promoting bacteria), were demonstrated through in vivo and in vitro assays. In vivo assays showed that *B. velezensis* 83 was able to control anthracnose (Kent mangoes) as efficiently as chemical treatment with Captan 50 PH™ or Cupravit hidro™. The inoculation of *B. velezensis* 83 to the roots of maize seedlings yielded an increase of 12% in height and 45% of root biomass, as compared with uninoculated seedlings. In vitro co-culture assays showed that *B. velezensis* 83 promoted *Arabidopsis thaliana* growth (root and shoot biomass) while, under the same experimental conditions, *B. velezensis* FZB42 (reference strain) had a suppressive effect on plant growth. In order to characterize the isolated strain, the complete genome sequence of *B. velezensis* 83 is reported. Its circular genome consists of 3,997,902 bp coding to 3949 predicted genes. The assembly and annotation of this genome revealed gene clusters related with plant-bacteria interaction and sporulation, as well as ten secondary metabolites biosynthetic gene clusters implicated in the biological control of phytopathogens. Despite the high genomic identity (> 98%) between *B. velezensis* 83 and *B. velezensis* FZB42, they are phenotypically different. Indeed, in vitro production of compounds such as surfactin and bacillomycin D (biocontrol activity) and γ-PGA (biofilm component) is significantly different between both strains. 
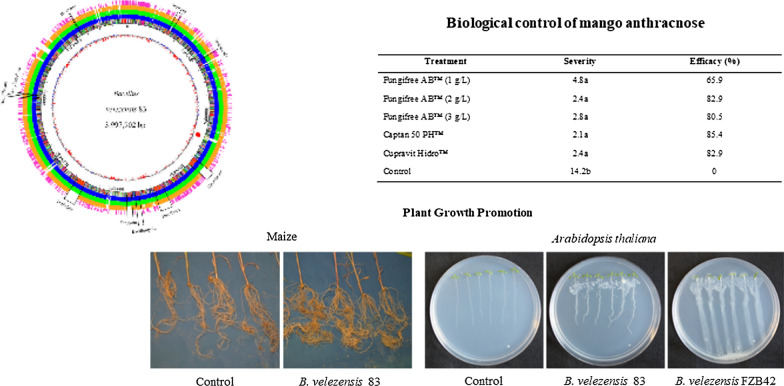

## Keypoints

*B. velezensis* 83 is a biological control agent and plant growth-promoting bacteria. Phylogenomic analysis allowed to reclassify* B. subtilis* 83 as* B. velezensis* 83.* B. velezensis *83 has genes to establish a rhizosphere plant-bacteria interaction.

## Introduction

*Bacillus* is a bacterial genus which comprises several species that establish, directly or indirectly, beneficial relations with plants (Olanrewaju et al. 2017). By direct mechanisms some *Bacillus* strains promote plant growth, by improving the acquisition of nutrients such as nitrogen and phosphate (biofertilization), or through the production of phytohormones (biostimulants) such as IAA (indol acetic acid), enzymes such as ACC deaminase (1-amyclocyclopropane-1-carboxylate deaminase) or volatile organic compounds (VOC) such as 2,3-butanediol and acetoin (Xu et al. 2014; Hanif et al. 2015; Asari et al. 2016, 2017; Vejan et al. 2016). On the other hand, through indirect mechanisms (production of antimicrobial compounds), they naturally exercise biological control over several phytopathogenic bacteria such as *Pseudomonas syringae*, *Agrobacterium tumefaciens*, *Xanthomonas campestris*, *Xanthomonas oxonopodis*, *Erwinia amylora* and fungal pathogens as *Botrytis cinerea*, *Fusarium oxysporum, Colletotrichum gloeosporioides, Rhizoctonia solani* and *Penicillium expansum* (Fira et al. 2018). *Bacillus* spp. biological control mechanisms include competition for nutrient and space, antibiosis or induced systemic resistance (ISR), which alone or together can reduce incidence and/or severity of plant diseases (Kloepper et al. 2004; Ongena and Jacques 2007; Choudhary and Johri 2009; Mongkolthanaruk 2012; Fan et al. 2018).

Genome sequencing strategies complemented with the analysis of secondary metabolite gene cluster profiles of several *Bacillus* strains have been helpful in order to identify potential biological control agents (BCA) or plant growth-promoting bacteria (PGPB); but also to estimate their disease prevention and benefical plant interaction potential (Borriss et al. 2011; Niazi et al. 2014; Palazzini et al. 2016; Cai et al. 2017; Belbahri et al. 2017; Fan et al. 2018; Chen et al. 2019). As part of the operational group *Bacillus amyloliquefaciens* (*B. amyloliquefaciens*, *Bacillus siamensis* and *Bacillus velezensis*), *B. velezensis* species has been recognized as a plant-associated bacteria due to the presence of nine gene clusters encoding enzymes involved in the nonribosomal synthesis of lipopeptides (surfactin, bacillomicyn D, fengicyn) and polyketides (macrolactin, bacillaene, difficidin), a siderophore (bacillibactin), a dipeptide (bacilysin) and a putative peptide with nematicide activity (Chen et al. 2007; Fan et al. 2017; Rabbee et al. 2019). Other genes involved in carbohydrate metabolism and plant cell wall degradation, such as amylase E (*amyE*), cellulase (*bglC*), xylanase (*xynACD*), galactokinase (*gal1*), and betaglucanase (*bglCS*) are also exclusively present in *Bacillus* strains associated to plants (Borriss et al. 2011). *B. velezensis* strains can show high genomic homology with phenotypic similarities, or differences, such as nutritional requirements, ability to colonize plants or production of antimicrobial metabolites (therefore antagonistic in vitro and in vivo activity) because they respond differently to stimuli from the habitat to which they are exposed. For example, the strains *B. velezensis* UCMB5044 (a oligotrophic PGPR isolated from desert soils), *B. velezensis* UCMB5113 and *B. velezensis* At1 (both strain plant endophytes) and *B. velezensis* UCMB55007 (a copiotroph isolated from calf gut) share 99% of genetic homology. However, their gene expression in response to root maize exudates were significantly different because they possess different DNA methylation patterns (Reva et al. 2019).

*B. velezensis* 83, is an aerobic, gram positive, rod shaped and spore forming bacteria isolated by our research team from the mango tree phyllosphere of orchards located in El Rosario, Sinaloa, México. This strain is the biological component of Fungifree AB™ marketed in México since 2012, as a foliar fungicide, very effective for the biocontrol of five different genera of phytopathogenic fungi (*Colletotrichum, Erysiphe, Botrytis, Sphaerotheca* and *Leveillula)* in crops of high agricultural importance such as mango, avocado, papaya, citrus, tomato, blueberry, blackberry, raspberry, zucchini, melon, cucumber, watermelon and others (Galindo et al. 2013). Here, we reported in vivo assays to demonstrate the activity of this strain as BCA (mango) as well as PGPB (maize). We have shown that *B. velezensis* 83 is an efficient strain for controlling mango anthracnose as well as a potent plant growth promotor agent in maize and *A. thaliana*. In addition, in vitro production by this strain of key compounds such as surfactin, bacillomycin D (biocontrol activity), acetoine and 2,3-butanediol, and particularly γ-PGA (biofilm component), was characterized, and helped to understand the mechanisms by which this strain exerts its action as a biofungicide and plant growth promotor. The genome sequence of *B. velezensis* 83, including genome assembly and annotation is also reported.

*B. velezensis* FZB42 was used as a reference for plant growth promotion and biological control strain (Fan et al. 2018). We show a comparison of metabolic and biological activities between *B. velezensis* 83 and *B. velezensis* FZB42 (> 98% genome identity) under in vitro conditions. However, as shown in this paper, a high genomic homology with a well characterized BCA strain is only the first step to fully characterize a newly isolate as BCA. The strain characterized in this work (*B. velezensis* 83), exhibited unique phenotypical traits in terms of biocontrol and growth promoting activities when compared with *B. velezensis* FZB42.

## Materials and methods

### Bacteria

*B. velezensis* 83 strain was deposited at Belgian Co-ordinated Collection of Micro-organisms (BCCM) by our research team under the accession number LMG S-30921. *B. velezensis* 83 was grown in 500 mL shake flasks containing 50 mL of Luria Bertani (LB) medium and incubated at 29 °C and 200 rpm for 12 h. Genomic DNA was isolated using a commercial kit (Quiagen). For the biocontrol and plant growth promotion assays, *B. velezensis* 83 was used as powder commercial formulation (Fungifree AB™ obtained from Agro&Biotecnia S. de R.L. de C.V.). *B. velezensis* FZB42 (BGSC 10A6, DSM23117) was kindly donated by Nord Reet UG Greifswald (Germany).

### Biological control and PGPB assays

A trial for biological control of mango anthracnose caused by *C. gloeosporioides* was done in a mango production orchard in El Rosario, Sinaloa, México. An experimental randomized block design was established with six treatments and four repetitions. The experimental unit was a mango Kent tree with more than 10 years old. In the experimental design, three *B. velezensis* 83 treatments (Fungifree AB™ in 1, 2 or 3 g/L), two chemical treatments (Captan 50™ in 300 g/L, and Cupravit hidro™ in 400 g /L) and a control (without treatment) were included. The treatments were applied to the foliage with a motorized-spray backpack. Four liters of the corresponding treatment were applied once a month in each tree from the beginning of flowering to the harvest of the fruits (six applications in total). Twelve fruits were taken from each experimental unit and stored (under commercial conditions) for 21 days, then the severity of anthracnose at postharvest was evaluated. The evaluation of severity and control efficacy obtained with each treatment was done using a visual hedonic scale, where: (0) healthy fruit, (1) < 2 mm spots, (2) < 5%, (3) < 12.5%, (4) < 25%, (5) < 50% and (6) > 50% of affected area in the mango fruit. The data collected were transformed to obtain the percentage of severity by means of the Eq. 1. (Townsend and Heuberger 1943):

1 $${\varvec{P}}=\left[\frac{\sum n.v}{N.C}\right] x 100$$

where:

P = severity (%).

*n* = number of samples per category.

*v* = numerical value of the category.

*N* = total number of samples.

*C* = highest category.

After that, the control efficacy of each treatment was calculated by Eq. 2. (Abbott, 1925):

2 $${\varvec{E}}{\varvec{f}}{\varvec{f}}{\varvec{i}}{\varvec{c}}{\varvec{a}}{\varvec{c}}{\varvec{y}}\boldsymbol{ }(\boldsymbol{\%})=\left[ IT-\left(\frac{it}{IT}\right) \right] x 100$$

where:

IT = severity (%) in the control.

it = severity (%) in the treatment.

### Plant growth promoting assays

Maize growth promotion study was carried out under greenhouse conditions. First of all, the seeds were disinfected by immersion in 1% NaClO aqueous solution for 1 min followed by three rinses with sterile distilled water. *B. velezensis* 83 was tested with two different treatments: 1) as a seed treatment or 2) as root treatment of seedlings. For seed treatment, the seeds were immersed in Fungifree AB™ aqueous solution (using 10 g/kg seed) during 2 h then sown in sterile substrate (peat moss: black earth, 3:1). For root treatment, Fungifree AB™ (at a dose equivalent to 2.5 kg/ha) was applied to each pot seedling at 10 and 22 days after sowing (DAS). For each treatment, 30 seeds or seedlings were used. For the control treatment, only distilled water was applied and the same quantity of seeds or seedlings was used. The evaluation of length shoot, dry weight (DW) of shoot and root was carried out with a sample of 15 plants at 37 DAS.

Another assay to show the plant growth promoting effects caused by *B. velezensis* 83 was done with in vitro co-cultures with *A. thaliana* (Col-0) seedlings and comparing with those effects caused by *B. velezensis* FZB42 and against a control (uninoculated plants). The seeds were disinfected and sown in aseptic conditions to obtain seedlings (Barrera-Ortiz et al. 2018). *A. thaliana* seedlings of 4 days after germination (DAG) were transferred to fresh agar plates with 0.2 × MS media inoculated with an aliquot of 10 µL (1 × 10^5^ cfu/mL) from an overnight culture of *B. velezensis* 83 or *B. velezensis* FZB42 (Fan et al. 2018). Six seedlings in each plate were carefully placed over the bacterial stria and whose shoots were approximately 1 cm from the bacterial inoculum. For Col-0 seedlings, the plates were incubated for an additional 6-day period and the root and primary root length, lateral root length (of the longest lateral root in each seedling) and shoot diameter were measured with a ruler, while lateral roots in the primary root and leaves in the shoot were recorded using a stereomicroscope Olympus SZ40 (Olympus Iberia S.A.U, Barcelona, España) at a 10X magnification. Lateral root density was calculated dividing lateral root number between the primary root length, and those parameters were obtained of 18 individuals for each treatment. Plant biomass, total fresh weight, shoot fresh weight and root fresh weight of 6 seedlings grown on the same plate were measured with an analytical balance Ohaus PA224 (Ohaus Corporation, Newjersey, USA), and three plates of each treatment were evaluated.

### Secondary metabolites production

For secondary metabolites production, *B. velezensis* 83 and *B. velezensis* FZB42 were grown in liquid batch cultures using a mineral medium with the following composition (in g/L): glucose 30.0, (NH_4_)_2_SO_4_ 6.0; K_2_HPO_4_ 7.98; KH_2_PO_4_ 9.6; MgSO_4_·7H_2_O 0.4; CaCl_2_ 0.1; FeSO_4_·7H_2_O 0.08; MnCl2·4H_2_O 0.019. The batch cultures of each strain were carried out inoculating 5 mL of an overnight culture in YPG medium (2 × 10^9^ cfu/mL) in 500 mL shaken flasks with 100 mL of working volume and incubated at 30 °C and 200 rpm for 48 h. Cultures were conducted at initial pH of 6.8 adjusted with NaOH before sterilization. *B. velezensis* FZB42 liquid batch culture was carried out under the same experimental conditions. Cell concentration (cell/mL) was determined using a Neubauer chamber.

#### Glucose, acetoin and 2,3-butanediol concentrations

A 1 mL sample was collected from the shaken flask and bacterial cells were removed from the medium by centrifugation (10,000 × *g*, 15 min) and filtration through a 0.2 µm membrane (hydrophilic and nonpyrogenic, Sartorious AG, Goettingen, Germany). Glucose, acetoin and butanediol in the supernatant were analyzed by high resolution reverse phase high-performance liquid chromatography (HPLC) using methodology reported previously (Cristiano-Fajardo et al. 2019) with minor modifications. Briefly, 20 µl of the sample was loaded to an Aminex HPX-87H column (7.8 × 300 mm; Bio-Rad Laboratories Inc., California, USA) and separated by using a Waters 2695 HPLC system (Waters Corporation, Massachusetts, USA). Acetoin was determined by absorbance at 210 nm and glucose and butanediol by refraction index. H_2_SO_4_ 5 mM was used as mobile phase with a flow of 0.6 mL/min. Column temperature was adjusted at 50 °C. Pure glucose, acetoin and butanediol (Sigma Chemical, St. Louis, MO, USA) were used as standards.

#### Surfactin and bacillomycin concentration

A 1 mL sample was collected from the shaken flasks and bacterial cells were removed from the medium as described above. The supernatant was loaded to a ZorbaxSB-C18 column (4.6 mm × 150 mm; Agilent Technologies, Palo Alto, CA, USA) and separated by HPLC by using a Waters 2695 HPLC system. The mobile phase was composed by 0.1% trifluoroacetic acid (TFA) in water (phase A), and 0.1% TFA in acetonitrile (phase B). Samples were eluted for 14 min using 40% of phase B at 32 °C with a flow rate of 0.3 mL/min, followed with a linear gradient of 40–85% for 1 min and maintained at 85% for another 36 min; finally a new linear gradient of 85–40% for 1 min, continued at 40% for 8 min. The elution pattern was monitored by determining absorbance at 205 nm. Pure surfactin and iturin (Sigma Chemical, St. Louis, MO, USA) were used as standards.

#### Poly-γ-glutamic acid concentration and mean molecular weight

Concentration of γ-PGA and mean molecular weight (MW) in the supernatant was determined by gel permeation chromatography (GPC) using methodology reported previously (Cristiano-Fajardo et al. 2019). Summarizing, 100 µl of cell-free supernatant was loaded into serial connected Ultrahydrogel columns (UG500/Linear; Waters Corporation, Massachusetts, USA), using a Waters 2695 HPLC system. γ-PGA was eluted with 0.1 M NaNO_3_ using a flow rate of 0.8 mL/min, columns temperature was adjusted at 38 °C. γ-PGA was detected with a refractive index detector (Waters 2414, USA). Pure γ-PGA (Sigma-Aldrich Inc., Missouri, USA) solutions were used as standards for polymer concentration calculation and poly(ethylene oxide) (Waters Corporation, Massachusetts, USA) with MWs in the range of 24–933 kDa were used as standards for mean MW.

### Genome sequencing, assembly an annotation

Genomic DNA from *B. velezensis* 83 was sent to MOgene Genome Sequencing Services (Mogene LC; St. Louis, MO, USA) with the following requests: libraries of 350 bp fragment size and paired end sequencing (2 × 250 bp) in MiSeq sequencer. The total number of reads was about ~ 7,000,000 paired reads that represents a genome coverture ~ 400X. Genome assembly was performed as described previously in Pérez-Carrascal et al. (2016). Briefly, a combined de novo and reference-based assembly was obtained with the Spades Genome Assembler (SPAdes; Bankevich et al. 2012). First, the SPAdes contigs were aligned, using NUCmer (Kurtz et al. 2004), to the complete genome of *B. velezensis* YAU-B9601-Y2 the closest phylogenetic relative to *B. velezensis* 83 (Hao et al. 2012). Second, the Illumina reads were mapped onto the contigs assembly, oriented according the 5´-3´directions of their pair-ends, and joined manually with Consed (Gordon et al. 2013). At the end, we obtained a single contig representing a closed circular chromosome 3,997,902 bp length. The protein-coding regions (ORFS, open reading frames) were predicted with Glimmer 3.02 (Delcher et al. 2007). The ORFs model was uploaded into Artemis 12.0 (Carver et al. 2012) to make ORFs frame rectifications and register manual annotations. Functional descriptions of genes were obtained by BlastX searches of the complete set of ORFs against the non-redundant database of the GenBank (Benson et al. 2008). A best-blast hits table was created with the parameters of the percentage of identity and similarity, coverture of the ORFs, coordinates of the matches, and the annotated function of the protein. Additional comparisons with the Conserved Domain Database (CDD) of the GenBank (Marchler-Bauer et al. 2015), Interpro (Mitchell et al. 2015), and IS-database (Siguier et al. 2006), contributed to confirm the GenBank-based annotations and to solve controversial cases. COG annotation was done using the NCBI COGs database (Tatusov et al. 2000) with BlastP comparisons (https://blast.ncbi.nlm.nih.gov) with the minimal similarity of 30% and e-value < 1 X10^−6^. The whole genome sequence of *B. velezensis* 83 was deposited in GenBank under the accession number CP034203.

### Genome analysis

Genome analysis was performed following the standard methodology described in González et al. (2019). In short, the pangenome model was obtained with the Bacterial Pangenome Analysis program (BPGA), by setting the USEARCH clustering algorithm to the default values (the minimal identity of 50% and 20 combinations) (Chaudhari et al. 2016). The phylogenetic tree was done in MEGA-6 evolutionary analysis software (Tamura et al. 2013) by Maximum Likelihood (ML) method using the JTT matrix (Jones et al. 1992) and bootstrap of 1000 replicates.

The accessory genome obtained with BPGA, was represented in a heatmap of presence and absence of genes and schematized with heatmap.2 (R´s gplots package; https://rdrr.io/cran/gplots). Genome comparisons were illustrated by circular maps obtained with GenVision of DNASTAR (Lasergene Core Suite (https://www.snapgene.com). Whole-genome comparisons were performed with selected *Bacillus* strains using Average Nucleotide Identity (ANI) calculated with JSpecies (Richter et al. 2016), and MUMmer 3.06 (Kurtz et al. 2004). Prophage searches were done with the Phaster program (Arndt et al. 2016).

### Statistical analysis

All data were analyzed by one-way analysis of variance (ANOVA) and Tukey comparison procedure assuming equal variances using Minitab™ 17 Statistical Software (Minitab, LLC, Pennsylvania, USA).

## Results

### *B. velezensis* 83 is a biological control agent and plant growth-promoting bacteria

**Fig. 1:**
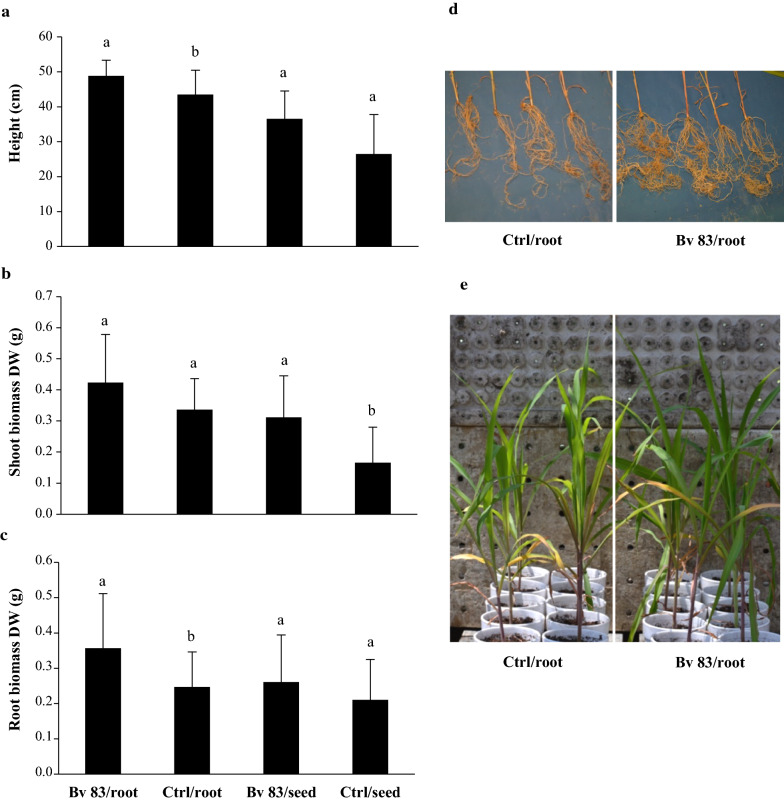
Growth-promoting effect of *B. velezensis* 83 on maize seedlings. **a** Height (cm) of the seedlings, **b** Shoot biomass (g) dry weight (DW) of the seedlings, **c** Root biomass (g) dry weight (DW) of the seedlings. Bv 83/root: Fungifree AB™ treatment to the root, Ctrl/root: without treatment to the root, Bv 83/seed: Fungifree AB™ treatment to the seed, Ctrl/seed: without treatment to the seed. **d** Aspect of the root of seedlings of Ctrl/root and Bv 83/root treatment. **e** Aspect of the shoot of seedlings of Ctrl/root and Bv 83/root treatment. N = 15. Different letters indicate statistically significant differences among treatments at *P* ≤ 0.05

Mango anthracnose severity was reduced in trees treated with *B. velezensis* 83 at three different doses, and fruit damage was similarly to those fruits harvested from trees with chemical conventional treatments (Captan 50 PH™ or Cupravit hydro™). The highest anthracnose severity in Kent mango fruits was found in the control treatment (Table 1). In maize, the application of *B. velezensis* 83 to the root caused an increase of 12% in height (Fig. 1a) of the seedlings, however, it has no effect on their shoot biomass (Fig. 1b) but an increase of 45% of the root biomass (Fig. 1c) of the seedlings was observed with respect to the uninoculated control. When the treatment was applied to seeds, the growth-promotion capacity of *B. velezensis* 83 was also evident. With respect to the control, the application of *B. velezensis* 83 caused an increase of 38% of the height and 88% of the shoot biomass, even if no effect on the root biomass was observed.

**Table 1:** Severity and biocontrol efficacy of *B. velezensis* 83 in mango ocharchs

Treatment	Severity	Efficacy (%)
Fungifree AB™ (1 g/L)	4.8a	65.9
Fungifree AB™ (2 g/L)	2.4a	82.9
Fungifree AB™ (3 g/L)	2.8a	80.5
Captan 50 PH™	2.1a	85.4
Cupravit Hidro™	2.4a	82.9
Control	14.2b	0

Different letters indicate significant differences among the treatments at *P* ≤ 0.05

### Genomic and pangenomic features of B. velezensis 83

**Fig. 2:**
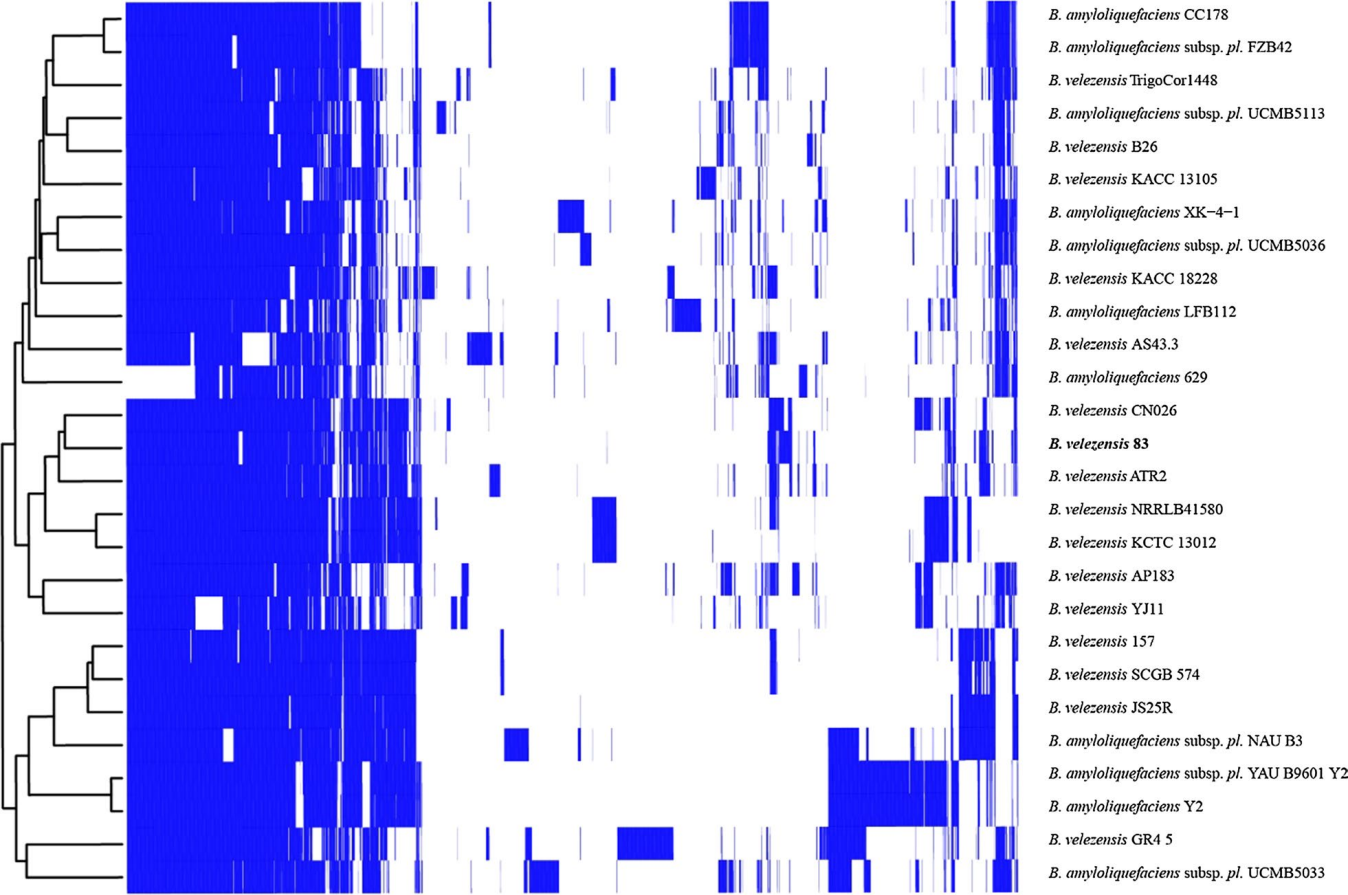
Accessory genome profile of *B. velezensis*. The heat-map indicates the presence (blue) and absence (white) of the accessory genes in 27 *B. velezensis* strains obtained from the BPGA pangenome model (Chaudhari et al. 2016). *B. velezensis* 83 is in bold letter

The complete genome of *B. velezensis* 83 consists of 3,997,902 bp with an average G + C content of 46%, 7 copies of the rRNAs operon (16S, 23S and 5S RNA) and 68 tRNA genes. The genome was predicted to encode 3752 coding sequences (CDS) of which 3255 were functionally annotated, whereas 497 were hypothetical. A total of 2892 CDS were assigned to COGs (cluster of orthologous groups). Functional classes defined by COGs indicate that *B. velezensis* 83 harbor a high proportion of proteins involved in carbohydrate (COG G) and amino acids transport and metabolism (COG E), as well as in transcription (COG K). The pangenome model of 27 selected strains of *B*. *velezensis* (including *B. velezensis* 83) and *B*. *amyloliquefaciens* strains approaches an asymptote (Additional file 1. Fig. S1) indicating, as in other *Bacillus* species, that *B*. *velezensis* has a closed pangenome structure and limited variation in gene content. The pangenome size consist of 5263 gene families, of which 2683 belong to the core genome, 1928 gene families in the accessory component, and a total of 652 unique gene families unevenly distributed in the 27 *B. velezensis* strains. Particularly, only 12 genes were unique in *B. velezensis* 83, most of them were hypothetical. The accessory genome described by a heatmap of presence/absence of genes indicates that although *B. velezensis* belongs to a clade with very related strains, each one still has some genetic differences that make each strain unique (Fig. 2).

### Taxonomic affiliation of strain 83

**Fig. 3:**
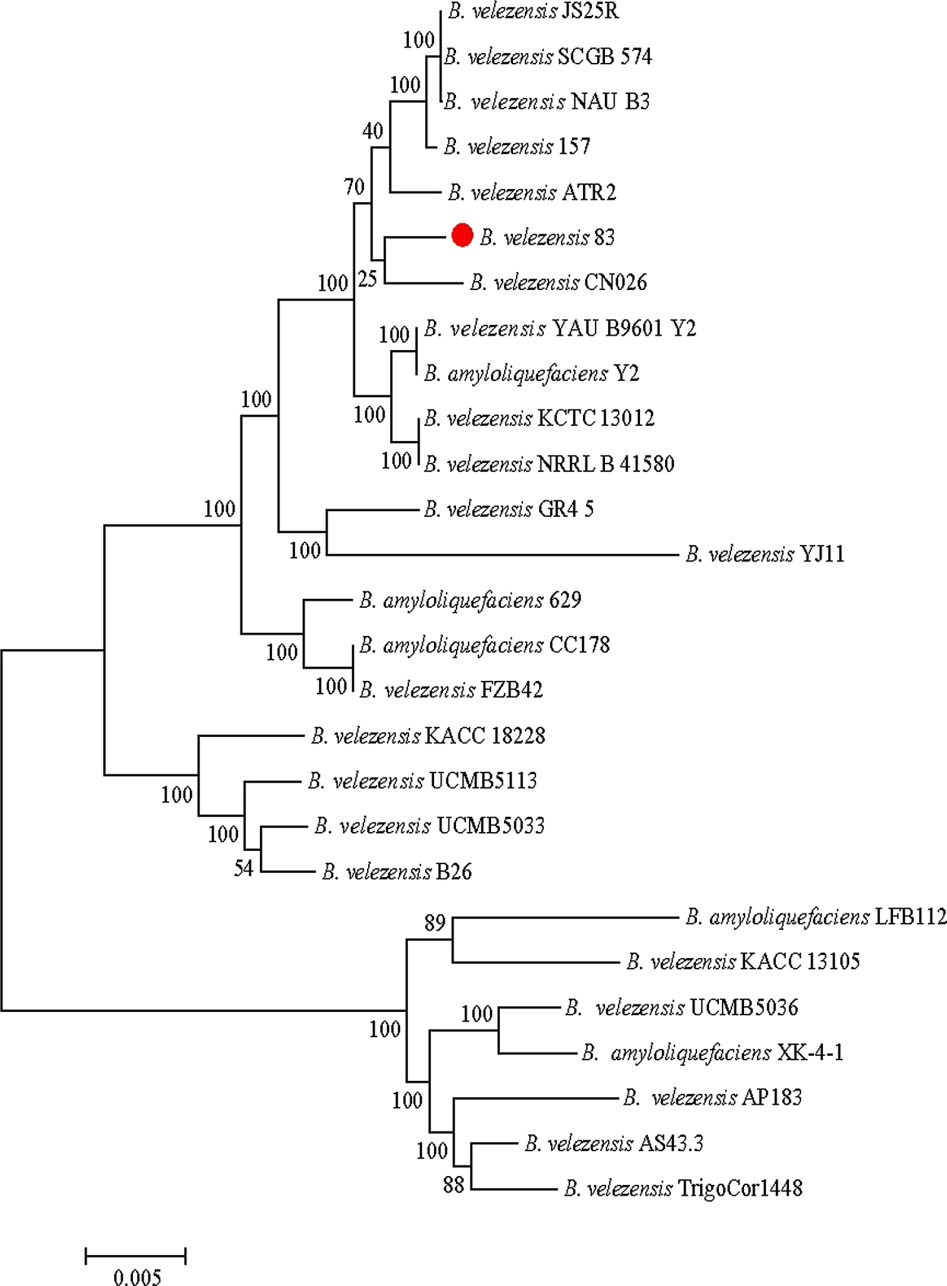
Taxonomic affiliation of *B. velezensis* 83. The phylogenomic tree was performed with 20 concatenated genes of the core families determined with BPGA (Chaudhari et al. 2016; see methods). *Bacillus* strains were selected from the phylogenomic neighbor joining tree reported in Dunlap et al. (2015). The tree is the result of 1000 replicates using the maximum likelihood method (ML) in MEGA program (Tamura et al. 2013). Red dot indicates the position of *B. velezensis* 83

Based on phylogenetic analysis of the 16S ribosomal genes, *Bacillus* strain 83 was initially classified as *Bacillus subtilis* (Galindo et al. 2013). To review this classification in the light of genome sequence presented here, we performed pairwise whole-genome comparisons between this strain and other *Bacillus* species using the Average Nucleotide Identity (ANI) (Richter et al. 2016). The results indicate that *B. subtilis* 83 is closely related to *B. velezensis* (ANIm > 97%, Additional file 1. Fig. S2). To support this observation, we did a phylogenomic analysis using 20 core housekeeping genes determined from the pangenome analysis of 27 *B. velezensis* strains (including strain 83). The phylogenetic tree, located the *B. subtilis* 83 together strains of *B*. *velezensis*, *Bacillus methylotrophicus*, and *B. amyloliquefaciens* subsp. *plantarum*, into a very related clade forming the recently recognized species called *B. velezensis* (Fig. 3) (Dunlap et al. 2015; Fan et al. 2017). Although *B*. *velezensis* and *B. amyloliquefaciens* are known plant growth-promoting bacteria, strains of the former are commonly associated with plants while the latter is constituted by soil-borne strains (Fan et al. 2017). These results support place *B. subtilis* strain 83 within *B. velezensis* species and allow us to correct its previous wrong classification*.*

### Genes related to biological control and sporulation

**Fig. 4:**
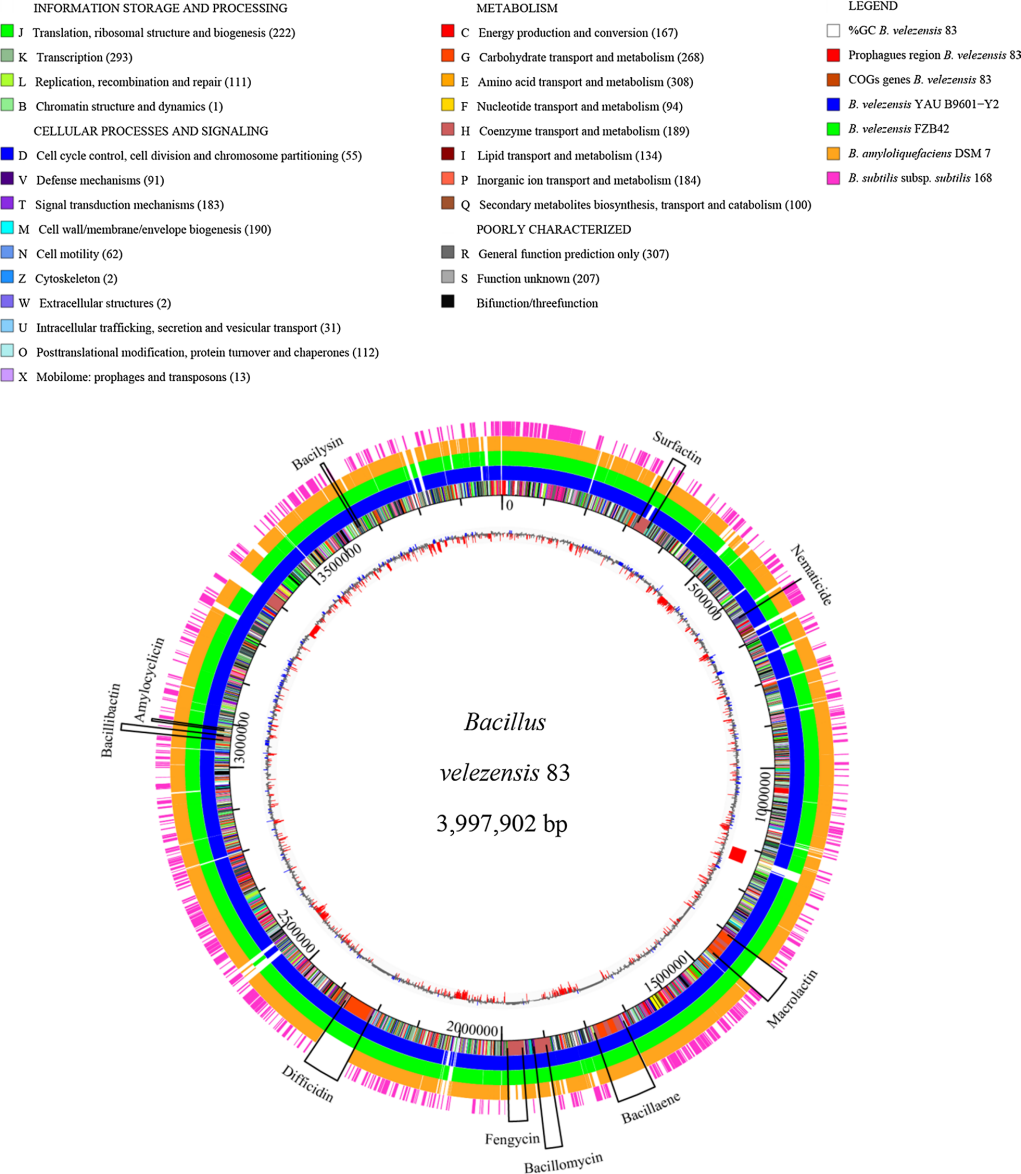
Graphical representation of *B. velezensis* 83 genome. From the innermost circle to the outermost: average GC (%) content profile, prophage region (red), the complete genome of *B. velezensis* 83. Color bars in the circle indicate the corresponding COG classification showed in the upper part of the scheme, the number of protein identified for each functional class is shown in (parentheses); subsequent circles indicate conserved regions with the genome of *B. velezensis* YAUB9601-Y2 (blue), *B. velezensis* FZB42 (green), *B amyloliquefaciens* DSM7 (orange), *Bacillus* subsp. *subtilis* (purple). The distribution of metabolite gene clusters is indicated by black rectangles and the cluster name

Genomic comparisons of *B*. *velezensis* 83 with the strains *B. velezensis* FZB42, *B. velezensis* YAUB601-Y2, *B. amyloliquefaciens* DMS7 and *B. subtilis* 168, showed high conservation and collinearity in the chromosomal sequence (Fig. 4). The genome of *B. velezensis* 83 harbors ten gene clusters dedicated to the synthesis of biocontrol metabolites (Table 2) with high genomic homology with *B. velezensis* FZB42 (Additional file 1. Table S1), covering about 8.2% of its genome. The genome of *B. velezensis* 83 contains five Non-Ribosomal Peptide Synthetases (NRPS) and three Polyketide Synthases (PKS) gene clusters. The *sfp* gene coding for the 4′-phosphopantetheinyl transferase responsible for the conversion of the apo-ACP domains of PKS and NRPS to their active holo-forms, was also identified in this strain together with the regulatory gene *yczE*. The predicted products of these NRPS gene clusters are the lipopeptides surfactin (*srfA*), bacillomycin (*bmy*) and fengycin (*fen*), the siderophore bacillibactin (*dhb*) and the dipeptide bacilysin (*bac*). In the PKS gene cluster, the genes encoding for macrolactin (*mln*), bacillaene (*bae*) and difficidin (*dfn*) were identified. In addition, a gene cluster probably involved in the production of the lantibiotic amylocyclicin, and the *pur* gene cluster for synthesis of a nematicide compound were located (Xia et al. 2011). However, *B. velezensis* 83 showed some incomplete gene clusters. For instance, the cluster for subtilin (*spa*) synthesis presents only five genes (*spaEFGRK*) out of ten reported; lacking those genes involved in the synthesis and transport of subtilin (Stein et al. 2002). The gene cluster for mersacidin (*mrs*) synthesis was also partially present, as only five genes (*mrsK2*, *mrsR2*, *mrsFGE*) out of ten reported were found; the genes for synthesis, modification and export of mersacidin are absent, keeping only those for regulation and immunity for this antibiotic (Schmitz et al. 2006). The results indicate that *B. velezensis* 83 may produce a wide repertoire of metabolites with biocontrol properties. The sporulation capacity of *Bacillus* has been exploited by the biopesticide industry to the preparation of powder formulations because spores are easier to handle and store, presenting a longer shelf life compared to liquid preparations. The sporulation process of *Bacillus* strains is carried out through the Spo0A pathway (Romero 2013; Liaqat et al. 2013; Yan et al. 2016) and the lack of some genes for the signaling cascade can affect the sporulation process (Branda et al. 2001; Yan et al. 2016). As predictable, in the genome of *B. velezensis* 83, all the genes involved in the different stages of the sporulation process (Additional file 1. Table S2), described by other authors (Romero 2013; López and Kolter 2010; Tan and Ramamurthi 2013) were found. Ten genes coding for Rap (response regulator aspartate phosphatase) proteins (Rap A1,-A2,-B,-C,-D,-F,-H,-J,-K1 and -K2) and three genes coding for Phr peptides (PhrA,-C-K) were identified. The interaction between Rap protein-Phr peptide results in a regulatory system which function is being the communication bridge between sporulation and competence process in *B. subtilis* (Schultz et al. 2009), the competence is a physiological state through the cell uptakes of exogenous DNA through which genomic diversity and evolution is generated (Brito et al. 2018).

**Table 2:** Genes involved in synthesis of biocontrol metabolites in *B. velezensis* 83

Metabolite	Gene cluster	Size (kb)	Position
Surfactin	*srfAABCD*	26.1	320,917–347,075
Macrolactin	*mlnABCDEFGHI*	53.2	1,404,749–1,458,017
Bacillaene	*baeBCDE*,*acpK*,*bacGHIJLMNRS*	71.7	1,714,735–1,787,205
Bacillomycin	*bmyCBAD*	36.9	1,890,815–1,928,064
Fengycin	*fenEDCBA*	37.6	1,950,976–1,988,645
Difficidin	*dfnMLKJIHGFEDCBXYA*	69.1	2,308,397–2,377,919
Amylocyclicin	*acnFEDCAB*	4.0	3,085,092–3,089,264
Bacillibactin	*dhbFBECA*	11.7	3,061,830–3,073,566
Bacilysin	*bacEDCBA*	4.7	3,661,180–3,665,866
Nematicide	*pur*(*EKBCSQLFMNHD*)	12.7	638,387–651,184

### Genes related to plant-bacteria interaction

*B. velezensis* 83 genome harbors genes involved in different process of plant-bacteria interaction (Additional file 1. Table S3). For biofilm formation, the operon for exopolysaccharide (*epsA-O*) synthesis and the operon *yqxM-sipW-tasA* for TasA protein fibers synthesis, are present (Al-Ali et al. 2018). Besides, genes coding for other biofilm components (Marvasi et al. 2010), such as the γ-polyglutamic acid polymer (*pgdS*, *pgsEACB*), the levansucrase (*sacB*) enzyme, and the proteases bacillopeptidase F (*bpr*), glutamyl endopeptidase protein (*mpr*), epr subtilisin family serine protease protein (*epr*), bacillolysin (*npr*) and the extracellular serine protease protein (*vpr*), were all found. Several genes exclusively present in *Bacillus* strains associated to plants were also found in *B*. *velezensis* 83 genome. That genes are involved in carbohydrate metabolism and plant cell wall degradation as described by Borriss et al. (2011), like amylase E (*amyE*), cellulase (*bglC*), xylanase (*xynACD*), galactokinase (*gal1*), and betaglucanase (*bglCS*). To assess the potential of *B. velezensis* 83 as plant growth-promoting bacteria, we also looked for genes already reported to promote the plant growth (Belbahri et al. 2017). Three different pathways described (Idris et al. 2007) for synthesis of the auxin indole acetic acid (IAA) were found: 1) the one of indole-3-pyruvate (IPyA) dependent of tryptophan transaminase gene clustering (*patB, YclC, YclB, DhaS*) products, 2) that of indole-3-acetonitrile (IAN) in which the nitrilase gene (*yhcX*) product acts, and 3) an uncharacterized IAA biosynthesis pathway in which the product of the gene acetyltransferase (*ysnE*) participates. The gene coding the protein involved in auxin excretion (*ywkB*) was also found. In addition, the cluster (*alsDSR*) encoding a α-acetolactate synthase for the synthesis of acetoin and 2,3-butanediol was present. Interestingly, even though *B. velezensis* 83 was isolated from mango tree phyllosphere, the strain has all the necessary genes to establish a rhizosphere plant-bacteria interaction. Therefore, in addition to control foliar phytopathogens, *B. velezensis* 83 seems to have the potential to be a biological control agent for root phytopathogens, probably eliciting ISR and/or promoting root growth.

*Phenotypic differences between B. velezensis* 83 and *B. velezensis* FZB42.

**Fig. 5:**
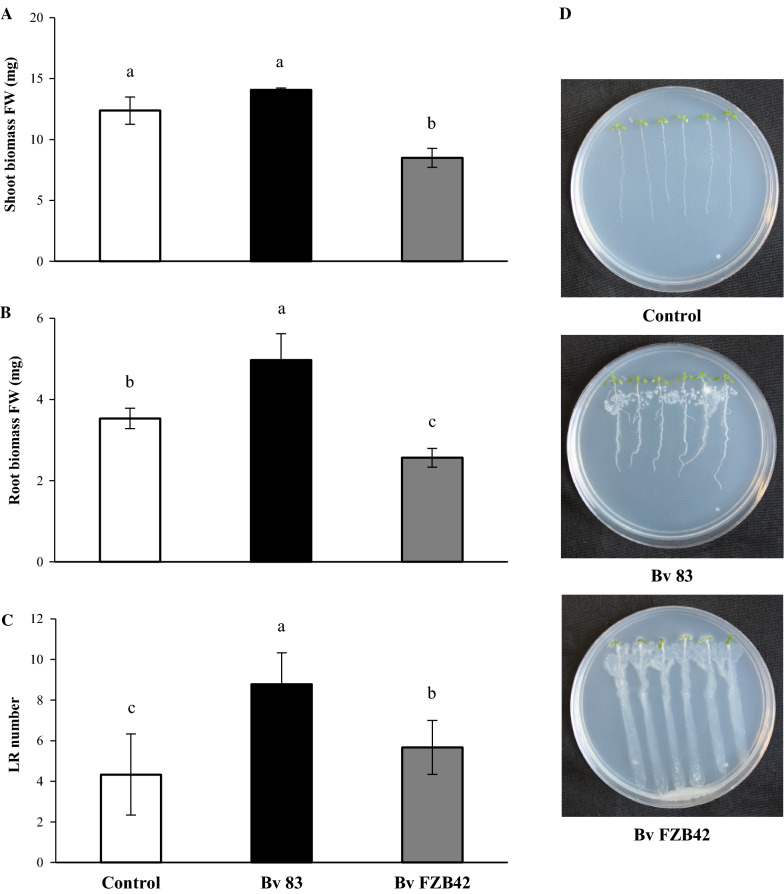
*B. velezensis* 83 and *B. velezensis* FZB42 growth-promoting effect on *A. thaliana* seedlings. **a** Shoot biomass (mg) fresh weight (FW). **b** Root biomass (mg) fresh weight (FW), **c** Lateral root (LR) number. N = 18. Different letters indicate statistically significant differences among treatments at *P* ≤ 0.05. **d** Aspect of control (not inoculated seedlings), *B. velezensis* 83 (Bv 83) and *B. velezensis* FZB42 (Bv FZB42) inoculated seedlings

*B. velezensis* 83 and *B. velezensis* FZB42 (reference strain) had  > 98% of identity (Additional file 1. Fig. S2). Through an in vitro co-culture assay with *A. thaliana* we showed the plant growth promotion effect of *B. velezensis* 83, it was compared with that caused by *B. velezensis* FZB42 and also both strains were compared against a control (uninoculated seedlings) (Fig. 5). After two days, *B. velezensis* 83 promoted the growth of *A. thaliana*, in contrast to the inoculation with *B. velezensis* FZB42, which had a suppressive effect over plant growth. There were no significant effects in terms of shoot biomass of *A. thaliana* caused by *B. velezensis* 83 inoculation (Fig. 5a); however, the main effect was observed in the root biomass (Fig. 5b). *B. velezensis* 83 increased significantly the lateral root number (102% more than the control) (Fig. 5c). This was similar to data reported for other *Bacillus* strains (Ryu et al. 2004, 2005; López-Bucio et al. 2007; Niazi et al. 2014; Asari et al. 2016, 2017; Verbon and Liberman, 2016; Islam et al. 2016; Kuan et al. 2016). Surprisingly, seedlings inoculated with *B. velezensis* FZB42 had only 69% and 73% shoot and root biomass (FW), respectively, as compared with the control. Furthermore, there were significant differences in other parameters of plant growth caused by both strains in *A. thaliana*: the primary root length, root density, lateral root length, shoot number, shoot diameter and total biomass were increased when using *B. velezensis* 83 but decreased when using *B. velezensis* FZB42 (Additional file 1. Fig. S3). Something characteristic of the inoculation of each strain was the bacterial pattern of seedling colonization. It was observed that *B. velezensis* 83 colonized forming a well defined and robust biofilm following the contour and development of the roots of each seedling, while *B. velezensis* FZB42 had an exacerbated growth and colonized extending its biofilm beyond the root and also invading the seedling shoot (Fig. 5d).

**Fig. 6:**
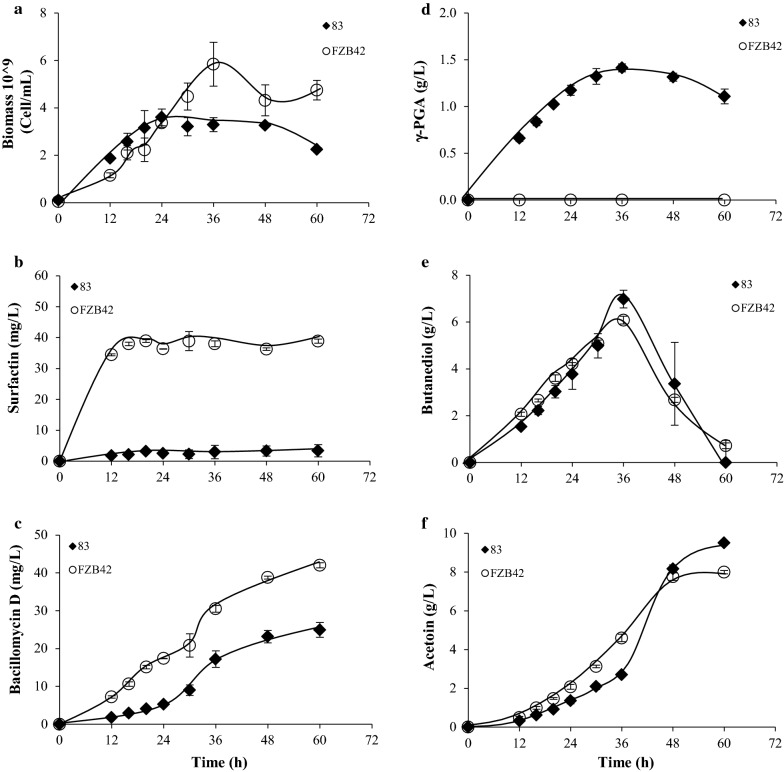
*B. velezensis* 83 vs* B. velezensis* FZB42 secondary metabolites production in liquid culture. **a** Biomass (cell/mL), **b** surfactin (mg/L), **c** bacillomycin (mg/L), **d** γ-PGA (g/L), **e** 2,3-butanediol (g/L) and **f** acetoin (g/L)

To test the functionality of some *B. velezensis* 83 gene clusters coding for synthesis of secondary metabolites related with biological control and plant interaction, several in vitro experiments were carried out (Fig. 6). With the culture conditions used in this study, *B. velezensis* 83 reached a maximum biomass of 3.3 × 10^9^ cell/mL (Fig. 6a), the stationary growth phase started after glucose and acetate were depleted in the medium (Additional file 1. Fig. S4). Surfactin and bacillomycin production was detected since 12 h of culture. *B. velezensis* 83 produced up to 3.4 mg/L of surfactin (Fig. 6b) and 23.2 mg/L of bacillomycin (Fig. 6c). The γ-PGA was produced between the 12^th^ and 36^th^ h of cultivation, the maximum γ-PGA concentration was 1.4 g/L (Fig. 6d) and it was associated with the increase of apparent viscosity (3.9–4.9 cp) in the media. The γ-PGA MW was 1.8–2.0 MDa, but this compound was consumed after glucose depletion in the culture media (Additional file 1. Fig. S4). The production of 2,3-butanediol and acetoin were associated with biomass growth and glucose metabolism; nevertheless, 2,3-butanediol was consumed when glucose was depleted in the media. The maximum 2,3-butanediol concentration was 6.97 g/L (Fig. 6e) and acetoin was 9.51 g/L (Fig. 6f). On the other hand, the strain *B. velezensis* FZB42 showed a similar behavior in glucose consumption, as well as acetate, 2,3-butanediol and acetoin production. Nevertheless, the main differences observed between *B. velezensis* 83 and *B. velezensis* FZB42 strains were the maximum growth, γ-PGA as well as lipopeptides production. With the culture conditions used in this work, *B. velezensis* FZB42 did not produce γ-PGA but instead produced more biomass (5.8 × 10^9^ cell/mL), and more surfactin (34.5 -39 mg/L since 12 h of culture time) and bacillomycin, than *B. velezensis* 83. We know that the gene cluster (*pgdS*, *pgsEACB*) involved in the synthesis γ-PGA are present in both strains *B. velezensis* 83 and *B. velezensis* FZB42 and they are of high identity (98%). Nevertheless, is known that *B. velezensis* 83 is a glutamic acid independent γ-PGA producing strain (Cristiano-Fajardo et al. 2019) as other few *Bacillus* strains (*B. subtilis* C10, *Bacillus licheniformis* A13, *B. licheniformis* TISTR 1010, *B. methylotrophicus*, *B. subtilis* NX2, *B. amyloliquefaciens* LL3) which only need glucose and NH_4_Cl as carbon and nitrogen sources, respectively, for γ-PGA synthesis (Cao et al. 2011; Hsueh et al. 2017; Sha et al. 2019). In contrast, lack of γ-PGA production by *B. velezensis* FZB42 could be explained due to a glutamate dependent mechanism. Therefore, *B. velezensis* 83 in addition to being a biological control agent and plant growth-promoting bacteria, is a new *B. velezensis* reported in the list of glutamate-independent γ-PGA producer strains reported recently (Sirisansaneeyakul et al. 2017).

A BlastP analysis (70% coverage with 50% identity) between *B. velezensis* 83 and *B. velezensis* FZB42 genomes shown that these two strains share 3475 core genes. In *B. velezensis* 83 genome there were 371 unique genes, 57% of them were genes with known function, 40% hypothetical proteins, 3% phage sequences and 1% kinase genes, while in *B. velezensis* FZB42 genome there were 262 unique genes. In *B. velezensis* 83 the *cysTWA* gene cluster (sulfate permease) and the *sbp* gene (sulfate ABC transporter substrate-binding protein) were found, these genes provide the ability to assimilating sulfite as well as thiosulfate to bacteria as sulfur sources (Guédon and Martin-Verstraete, 2006). Furthermore, *cysTWA*, *sbp* and *cysP* (thiosulfate transporter) genes complete the synthesis pathway of amino acid cysteine which has only been reported for the *B. subtilis* BD170 strain and other bacteria such as *Escherichia coli* and *Salmonella typhimurium* (Mansilla and de Mendoza, 1997). Cysteine is an amino acid present in antimicrobial peptides of ribosomal synthesis as bacteriocins (Abriouel et al. 2011) and other peptides as subtilisin (Graycar et al. 2013), surprisingly subtilin and mersacidin gene cluster were incomplete and only three genes for subtilisin (serine proteases: *apr*, *aprx* and *epr*) were found in *B. velezensis* 83 genome. Some *Bacillus* spp. strains also produce sulfure containing VOC such as carbon disulfide, dimethyltrisulfide and thiophene, which has been reported with antifungal activity (Caulier et al. 2019). Other relevant genes such as quorum-sensing (QS) pheromone *comX*, *rap* proteins (*rapK1*, *rapK2* and *rapH*) and *phrK* peptide were identified as unique genes. In *B. subtilis* group ComX pheromone determines the pherotype and that the social communication cell–cell to stablish community structures within and between biofilms (Kalamara et al. 2018). ComX is the first signal to activate the transcription factor ComA which regulates the expression of several genes involved in the sporulation-competence signal transduction network (Schultz et al. 2009) as well as the production of surfactin, exoproteases and extracellular matrix for biofilm formation (Kalamara et al. 2018). ComX also could be different for strains of the same specie as reported for *Bacillus* spp. strains isolated from rhizoplane of tomato plants, which also showed different growth promotion effect over the plants (Oslizlo et al. 2015). There are two groups of Rap proteins, those that show phosphatase activity (RapA, RapB, RapE, RapH and RapJ) over effector response regulators (RR) and those that exert their function blocking the DNA-binding activity (RapC, RapF, RapG, RapH, and RapK) of their RR target, each Rap protein is specifically inhibited by a Phr peptide (Gallego del Sol and Marina, 2013). RapH protein negatively regulates *srfA* expression in *B. velezensis* MT45 and *B. velezensis* DSM7 (Zhi et al. 2017) and RapK-PhrK regulates the expression of several genes known to be activated directly by ComA (competence-sporulation genes) and indirectly by Spo0A (sporulation-biofilm genes) (Auchtung et al. 2006).

## Discussion

*B. velezensis* 83 is an efficient biocontrol agent of mango anthracnose and plant growth-promoting bacteria of maize. México is one of the biggest worldwide mango exporters, for this reason Mexican mango orchards are worked carefully to obtain fruit with phytosanitary quality for its competitiveness in the international market. This means that mangoes pests (like fruit fly) and diseases (like anthracnose) must be absent at the harvest day as well as during the time of storage previous to its commercialization at the final export destination (21 days for boat transport from México to Japan). When chemical products are used for the control of anthracnose, it is important to ensure that the maximum residue limits (MRLs) of pesticides are under the maximal concentration allowed by international standard. Therefore, the relevance of biological control with *B. velezensis* 83 lies in the fact that it is an innocuous product for human consumption, whose application is efficient for the control of anthracnose in mango Kent (between 65 and 80%) such as treatments with agrochemicals (Captan 50 PH™ or Cupravit hydro™). *B. velezensis* 83 produces, in vitro*,* bacillomycin D, a lipopeptide that inhibits both spore germination and mycelial growth, propidium iodide staining of spores and mycelium showed that bacillomycin D caused damage to the cell membranes of *C. gloeosporioides*, affecting directly its viability (Luna-Bulbarela et al. 2018). Interestingly, *B. velezensis* 83 genome harbors three gene clusters coding synthases dedicated to the production of antifungal compounds, including the lipopeptide fengycin, the siderophore bacillibactin and the dipeptide bacilysin, which could contribute to its ability for the biocontrol of mango trees anthracnose. Other *Bacillus* strains have similar antimicrobial compounds production and biocontrol capacity. For example, a 70% of control for crown rot causing pathogens (*Lasiodiplodia theobromae, Thielaviopsis paradoxa, Colletotrichum musae* and *Fusarium verticillioides*) was obtained in postharvest banana fruits inoculated with *B. amyloliquefaciens* DGA14 (10^8^ cfu/mL) (Alvindia, 2013). *B. amyloliquefaciens* GYL4 (10^4^–10^8^ cfu/mL) displayed between 46–93% of control efficacy on anthracnose of cucumber (Kim et al. 2016), *B. velezensis* RC 218 (10^4^–10^6^ cfu/mL) (Palazzini et al. 2016) and *B. velezensis* TrigoCor1448 (> 10^8^ cfu/mL) (Crane et al. 2013) were able to control the *Fusarium* head blight in wheat.

*B. velezensis* 83 as plant growth-promoting bacteria had a different effect on the growth of the maize seedlings development depending on the phenological state of the maize, application to root seedling promoted the root development, in contrast application in seed state promoted the shoot development. Traits as phosphate solubilization, auxin and VOC (as 2,3-butanediol and acetoin) production by *B. velezensis* 83 could be involved in the growth promotion of maize as has been reported for other strains (Bentes et al. 2019; Cui et al. 2019). Plant hormone homeostasis is affected by PGPR production of auxins, ethylene, gibberellins, abscisic acid, salicylic acid, jasmonic acid, and different effects are observed on shoot or root system, being auxin the main hormone which regulates the plant growth and development (Tsukanova et al. 2017). PGPR production of auxins promotes plant-PGPR interaction, activates jasmonic acid dependent plant resistance, affects the expression of genes involved in auxin synthesis and transport (influx and efflux carriers), the PGPR colonization site affect the auxin gradient in the plant and also the bacterial VOC affect the auxin genes expression (Tsukanova et al. 2017). However, it has been reported that auxin has a complex crosstalk network (involving gene expression, signal transduction, and metabolic conversion process) with cytokinin and ethylene to coordinate the root development in *A. thaliana* (Liu et al. 2017a, b), thus the PGPR could affects several physiological processes in the plant at the same time.

In order to show the plant growth promotion effect, *B. velezensis* 83 was used in the in vitro assays with the model plant *A. thaliana* using 10^5^ cfu/mL for in vitro inoculation, because a higher concentration of *B. velezensis* 83 cells (> 10^7^ cfu/mL) had a growth suppressive effect on *A. thaliana* (data not shown). On the other hand, *B. velezensis* FZB42 in 10^3^ cfu/mL concentration of bacteria did not show sufficient growth in the stria to generate reproducible results on the *A. thaliana* seedlings (data not shown). The plant growth promotion activity by *B. velezensis* FZB42 on *Lemna minor* plantlets (grown in 48-well microtiter plates) was dependent on the concentration of bacterial culture filtrate (%) or the quantity of growing bacterial cells (cfu) added (Idris et al. 2007). The higgest stimulatory effect on *L. minor* plantlets was using 2 × 10^5^ cfu of *B. velezensis* FZB42, the result was attributed to the IAA production by this strain. Also, the authors reported that the lowest dilution of bacterial culture filtrates (0.1%) had a stimulatory effect on the seedling growth, but the highest concentration of bacterial growing cells (1 × 10^7^ cfu) added had a negative effect on plantlets growth (Idris et al. 2007). *B. velezensis* FZB42 (former *B. amyloliquefaciens* FZB42) inoculation (10^6^ cfu/mL) promoted the plant growth of *A. thaliana* under non-stress and saline conditions, the strain enhanced a 28.3% and 27.2% the shoot biomass dry weight at 0 and 100 mM NaCl compared with non-inoculated seedlings, respectively, nevertheless the effect on the root biomass dry weight was not considered in their evaluation. The inoculation of *B. velezensis* FZB42 increased the expression of auxin and photosynthesis related genes on *A. thaliana* (Liu et al. 2017a, b).

This study showed the capability *B. velezensis* 83 to synthetize in vitro antimicrobial compounds such as bacillomycin D, ISR elicitor compounds as surfactin, growth promotion compounds such as acetoin and 2,3-butanediol, and also the biopolymer γ-PGA (a biofilm component). The in vitro results of secondary metabolites production and *B. velezensis* 83-*A. thaliana* co-cultures assays suggest that the capacity of *B. velezensis* 83 to synthesize high concentration of γ-PGA and low concentration of surfactin (in contrast with *B. velezensis* FZB42) which contributed significantly to the pattern of biofilm formation on the root and resulted in a positive effect on seedling growth. The lipopeptides and polyketides compounds have the fundamental function of inhibiting plant pathogens (Mongkolthanaruk 2012); on the other hand, surfactin is a metabolite known for its antimicrobial activity, elicitor of the plant immune response and biofilm promoter (Ongena and Jacques 2007; Jourdan et al. 2009). Some components of the culture medium such as carbon, nitrogen and metal ion sources, were determinant of the level of production of antifungal activity of *Bacillus sp*. BH072 against *Aspergillus niger*, *B. cinerea* and *F. oxysporum* (Zhao et al. 2014). The γ-PGA could improve the abilities of *B. amyloliquefaciens* C06 cells to attach to smoot surfaces, to form biofilm and colonies, as well as to swarm on semisolid surface in vitro, is critical for increasing the robustness and complex morphology of the colony biofilm (Yu et al. 2016). *B. subtilis* γ-PGA producer cells had a higher root colonization efficiency (cfu of root-associated *B. subtilis* cells per gram of collected soil) than γ-PGA non-producers *B. subtilis* cells. Indeed, γ-PGA production improves colonization efficiency of *B. amyloliquefaciens* C06 on apple surface (Liu et al. 2010). In terms of VOC production, 2,3-butanediol and acetoin of *B. subtilis* GB03 and *B. amyloliquefaciens* IN937a, can reduce infection severity of *A. thaliana* seedlings by *Erwinia carotovora* subsp. *carotovora* strain SCC1 (Ryu et al. 2004). These VOCs also trigger plant growth promotion in *A. thaliana* (Farag et al. 2013). Several VOC structures produced by *B. amyloliquefaciens* UCMB5113 were identified by GC–MS analysis, different VOC profiles were found depending on the growth medium composition (Asari et al. 2016). On the other hand, acetoin and butanediol activated the abscisic and salicylic acid signaling networks in *A. thaliana* and *Nicotiana benthamiana* (tobacco), stimulating the production of nitric oxide and hydrogen peroxide during stomatal closure, which may prevent the plant from microbial infection (Wu et al. 2018).

Phenotypic differences between strains of high genetic homology may be caused by differences in the methylation patterns of the genes as a result the presence of different restriction-modification genes between the strains. For example, the type I restriction-modification DNA-methyltransferases hsdMSR was found in *B. velezensis* UCMB5113 and At1 strains and methyltransferase BamHIM-restriction enzyme BamHI complex, was uniquely identified in *B. velezensis* UCMB5007 and UCMB5044 strains. These methyltransferases can interfere in the sequences of the promoters in the genes (i.e. in the transcriptional regulator SigA which in turn regulates other sigma factors) which affects their expression and causes phenotypic differences between the strains when faced with environmental stimuli (Reva et al. 2019). In terms of gene expression, the *B. amyloliquefaciens* LL3 strain is a model because it is a glutamate-independent producer of γ-PGA and several mutants have been constructed. The mutant *B. amyloliquefaciens* LL3 Δ*pgsBCA* (non-producer of γ-PGA) showed a significant increase in the expression levels of the genes for synthesis of the antibiotics bacillaene, surfactin, iturin and fengicin (Gao et al. 2017). These authors suggest that the synthesis of these four antibiotics directly or indirectly affects the synthesis of γ-PGA and vice versa, in particular the synthesis of iturin and γ-PGA depends on the same substrate (glutamates or glutamines) and on the acetyl-coA of the TCA that is part of the synthesis machinery of both compounds. Also, the mutant *B. amyloliquefaciens* LL3 Δ*srf* (non-producer of surfactin), shown defects in biofilm formation and swarming, but increased γ-PGA production by ~ 24% more than *B. amyloliquefaciens* LL3 wild strain (Gao et al. 2017). *B. amyloliquefaciens* LL3 has the complete pathway for synthesis of iturin, however, the lipopeptide could not be detected in the culture medium also with the γ-PGA synthetase knockout strain NK-∆LP (non-producer γ-PGA), but *B. amyloliquefaciens* C2LP (derived from NK-∆LP) showed a considerable increase in the expression levels of the iturin synthesis genes due to the insertion of a constitutive promoter (C2up) in the iturin operon (Dang et al. 2019). *B. amyloliquefaciens* MT45 was able to achieve a tenfold surfactin production in comparison to the very close-related strain *B. amyloliquefaciens* DSM17 (Zhi et al 2017). The high production of surfactin in *B. amyloliquefaciens* MT45 was due to factors such as: a) the amount of ABC transporter proteins in the cell (as this contributes to detoxification of surfactin and to the assimilation of glutamate); b) the overexpression of genes related to nitrogen metabolism (important for amino acid synthesis); and c) changes in central carbon metabolism and lipid synthesis. *B. amyloliquefaciens* MT45 has eight encoding genes ABC transporters and two resistance-related genes and has been suggested that they favor the production of surfactin because they provide resistance to the cells against the antibiotic. On other hand, it has been shown that ~ 23% of the *B. amyloliquefaciens* FZB42 genes that showed changes in expression levels in the presence of maize plant root exudates (Fan et al. 2012), were genes of unknown function of which nineteen genes were unique to the strain and 15% were hypothetical or putatively functioning proteins. Therefore it is probable that the differences observed between *B. velezensis* 83 and *B. velezensis* FZB42 in this work about the production of surfactin, bacillomycin, γ-PGA, as well as in the formation of biofilm, are due to differences in gene regulation and expression related to the *srf*, *bmy* and γ-PGA gene clusters and also of unique genes as has been showed for other *Bacillus* spp. strains.

In conclusion, *B. velezensis* 83 is an efficient biocontrol agent of mango anthracnose and plant growth-promoting bacteria of maize and *A. thaliana*. *B. subtilis* 83 was reclassified as *B. velezensis* 83 according to a phylogenomic analysis. *B. velezensis* 83 genome harbors the genes to produce numerous secondary metabolites that are determinant for plant-bacteria interaction, sporulation, biocontrol and PGPB activity. *B. velezensis* 83 strain has all the necessary genes to establish a rhizosphere plant-bacteria interaction. Therefore, in addition to control foliar phytopathogens, *B. velezensis* 83 has the potential to be a biological control agent for root phytopathogens, probably eliciting ISR and promoting root growth. Our results are evidence that the *B. velezensis* 83 and *B. velezensis* FZB42 strains are phenotypically different, despite the fact that they have high genetic identity (> 98%), and it allows us to highlight the importance of the complementarity of the genomic sequencing with in vitro test and plant assays to identify *B. velezensis* 83 as a new effective strain for biological control, plant growth promotion and glutamate-independent γ-PGA production.

## Supplementary information

**Additional file 1:** **Figure S1.** The pangenome model of 27 selected strains of *B*. *velezensis* (including *B. velezensis* 83) and *B*. *amyloliquefaciens* strains. **Figure S2.** Whole-comparison of species and strains of *Bacillus*. Pairwise ANIm was calculated using JSpecies (Richter et al. 2016). **Figure S3.** *B. velezensis* 83 and *B. velezensis* FZB42 growth-promoting effect on *A. thaliana* seedlings. **Figure S4.** *B. velezensis* 83 vs *B. velezensis* FZB42 secondary metabolites production in liquid culture. **Table S1.** Comparative BlastP (70% coverage and 50% identity) analysis of secondary metabolites genes of *B. velezensis* 83 and *B. velezensis* FZB42. **Table S2.** Genes involved in bacterium sporulation. **Table S3.** Genes involved in plant-bacterium interactions.

## Data Availability

The datasets supporting the conclusions of this article are included withing the article and its additional files.
