# Supplementary material for: Bacillus velezensis 83 a bacterial strain from mango phyllosphere, useful for biological control and plant growth promotion
Source: AMB Express. 2020 Sep 7;10:163. doi: 10.1186/s13568-020-01101-8 (PMC7477031; doi:10.1186/s13568-020-01101-8)
Supplement: Supplementary file 1 — Additional file 1: Figure S1. The pangenome model of 27 selected strains of B. velezensis (including B. velezensis 83) and B. amyloliquefaciens strains. Figure S2. Whole-comparison of species and strains of Bacillus. Pairwise ANIm was calculated using JSpecies (Richter et al. 2016). Figure S3. B. velezensis 83 and B. velezensis FZB42 growth-promoting effect on A. thaliana seedlings. Figure S4. B. velezensis 83 vs B. velezensis FZB42 secondary metabolites production in liquid culture. Table S1. Comparative BlastP (70% coverage and 50% identity) analysis of secondary metabolites genes of B. velezensis 83 and B. velezensis FZB42. Table S2. Genes involved in bacterium sporulation. Table S3. Genes involved in plant-bacterium interactions. [file 13568_2020_1101_MOESM1_ESM.docx]

**AMB Express**

***Bacillus velezensis* 83 a bacterial strain from mango phyllosphere, useful for biological control and plant growth promotion**

**Karina A. Balderas-Ruíz^a^, Patricia Bustos^b^, Rosa I. Santamaria^b^, Víctor González^b^, Sergio Cristiano-Fajardo^a^, Salvador Barrera-Ortíz^c^, Miriam Mezo-Villalobos^d^, Sergio Aranda-Ocampo^e^, Ángel Arturo Guevara-García^c^, Enrique Galindo^a^ and Leobardo Serrano-Carreón^a*^**

**^a^ Depto. de Ingeniería Celular y Biocatálisis, Instituto de Biotecnología, Universidad Nacional Autónoma de México, Av. Universidad #2001, Col. Chamilpa, C. P. 62210, Cuernavaca, Morelos, México.**

**^b^ Centro de Ciencias Genómicas, Universidad Nacional Autónoma de México, Av. Universidad #2001, Col. Chamilpa, C. P. 62210, Cuernavaca, Morelos, México.**

**^c^ Depto. de Biología Molecular de Plantas, Instituto de Biotecnología, Universidad Nacional Autónoma de México, Av. Universidad #2001, Col. Chamilpa, C. P. 62210, Cuernavaca, Morelos, México.**

**^d^ Agro&Biotecnia S de RL de CV, Limones #8, Col. Amate Redondo, C. P. 62334, Cuernavaca, Morelos, México.**

**^e^Depto. Fitosanidad-Fitopatología, Colegio de Postgraduados, Carretera México-Texcoco Km. 36.5 Montecillo, Texcoco C.P. 56230, Edo. de México, México.**

*Corresponding author at: Departamento de Ingeniería Celular y Biocatálisis, Instituto de Biotecnología, Universidad Nacional Autónoma de México, Av. Universidad #2001, Col. Chamilpa, C.P. 62210 Cuernavaca, Morelos, México

*E-mail address*: leobardo@ibt.unam.mx (Leobardo Serrano-Carreón)

**Fig. S1** The pangenome model of 27 selected strains of *B*. *velenzensis* (including *B. velezensis* 83) and *B*. *amyloliquefaciens* strains

**
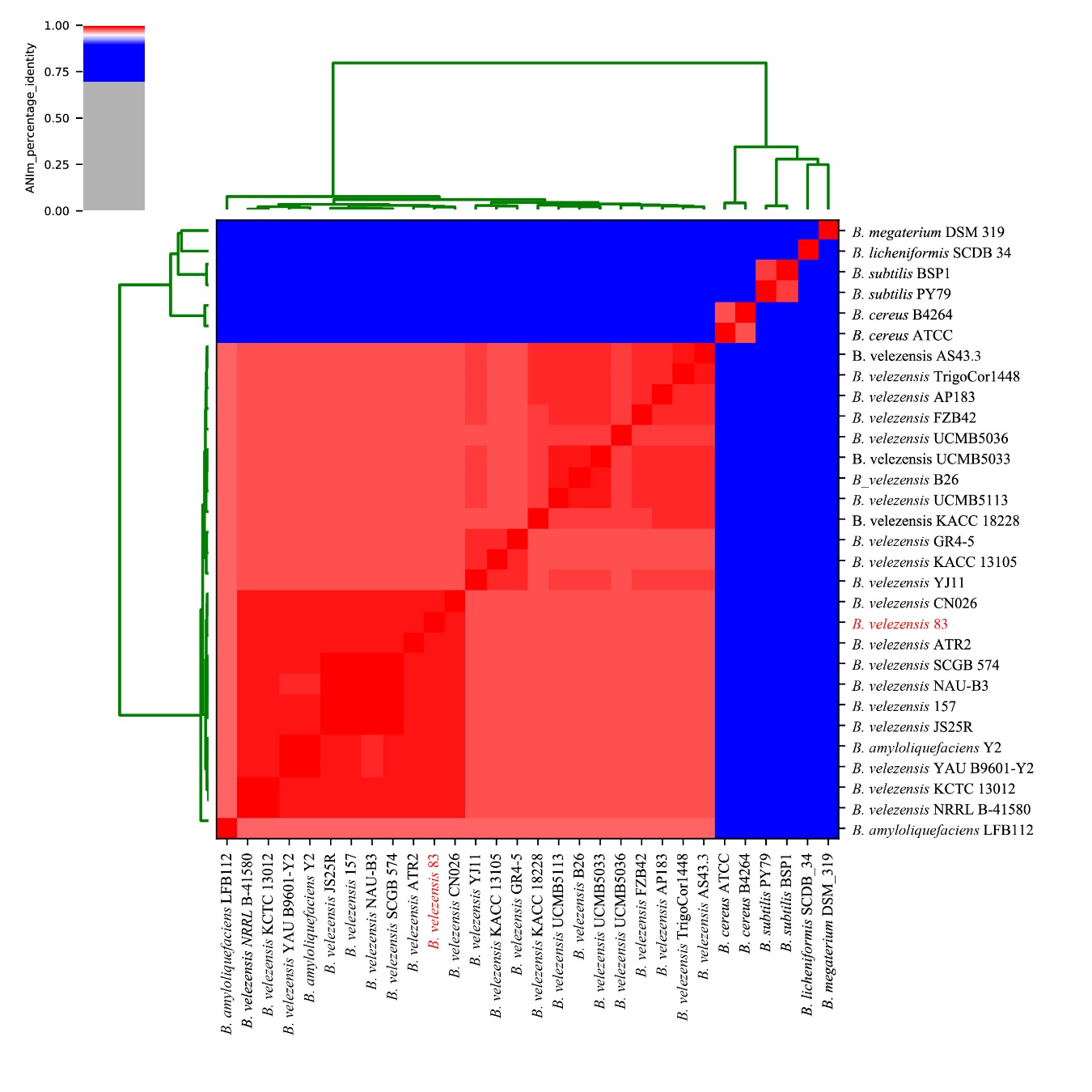
**

**Fig. S2** Whole-comparison of species and strains of *Bacillus*. Pairwise ANIm was calculated using JSpecies (Richter et al. 2016). The ANIm scale in the inset shows the highest identity ANI% >98% in red, and ANIm <95% in blue. *B. velezensis* species formed the largest group (in red) and include *Bacillus velezensis* 83. Outside of this group, in small red squares, are other *Bacillus* species. The original names of *Bacillus* strains and species are the same as in the GenBank


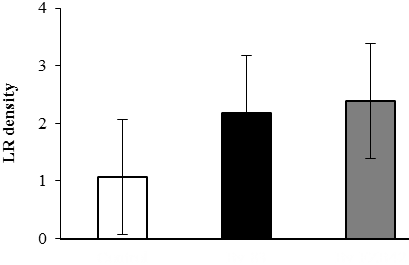

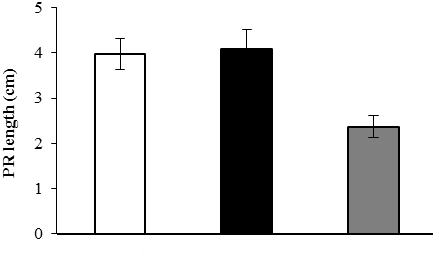

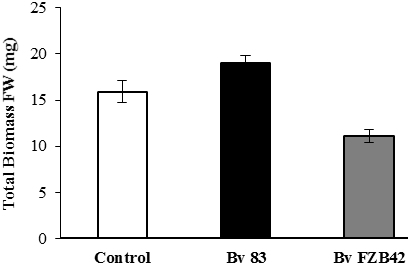

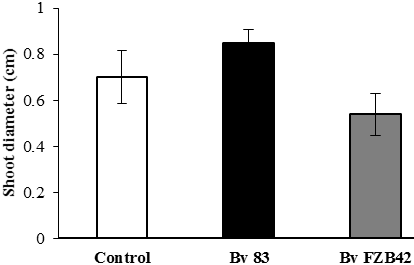

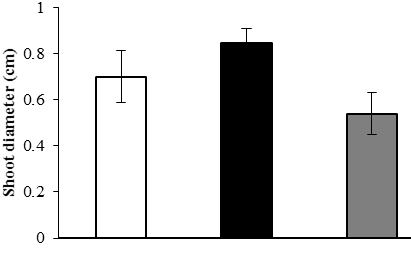

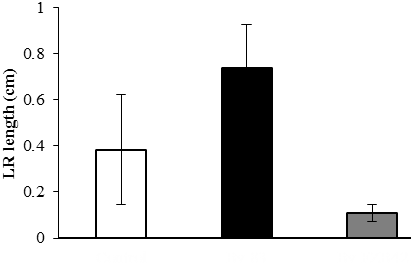


a

a

b

b

a

c

b

a

c

b

a

c

a

a

b

b

a

a

**c**

**b**

**a**

**f**

**e**

**d**

**Fig. S3** *B. velezensis* 83 and *B. velezensis* FZB42 growth-promoting effect on *A. thaliana* seedlings. a) primary root (PR) length (cm), b) lateral root (LR) length (cm), c) shoot diameter (cm), d) lateral root (LR) density, e) leave number, f) total biomass (mg). N=18. Different letters indicate statistically significant differences among treatments at *P*≤0.05

**a**

**c**

**d**


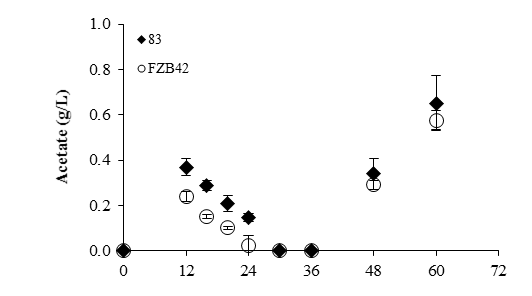


**Time (h)**


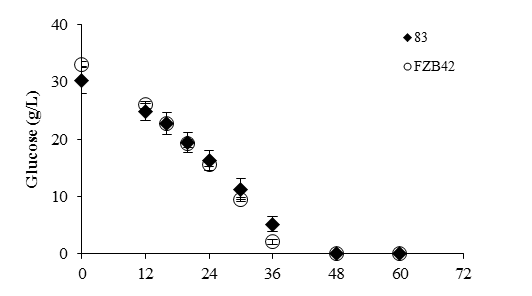


**b**

**Time (h)**

**Fig. S4** *B. velezensis* 83 (♦) *vs* *B. velezensis* FZB42 (○) secondary metabolites production in liquid culture. a) γ-PGA (Da), b) glucose (g/L), c) apparent viscosity (100 s-1, cp), d) acetate (g/L)

**Fig. S4** *B. velezensis* 83 *vs* *B. velezensis* FZB42 secondary metabolites production in liquid culture. a) γ-PGA (Da), b) glucose (g/L), c) apparent viscosity (100 s-1, cp), d) acetate (g/L)

**Table S1** Comparative BlastP (70% coverage and 50% identity) analysis of secondary metabolites genes of *B. velezensis* 83 and *B. velezensis* FZB42

| ***B. velezensis* 83 (locus/description)** | **Product length**  ***B. velezensis***  **83** | **Product length**  ***B. velezensis***  **FZB42** | **Identity** | ***B. velezensis* FZB42 (locus/description)** |
| --- | --- | --- | --- | --- |
| EBA29_RS01660 *srfAA* surfactin non-ribosomal peptide synthetase | 3584 | 3584 | 97% | RBAM_RS01835 *srfAA* surfactin non-ribosomal peptide synthetase SrfAA |
| EBA29_RS01665 *srfAB* surfactin non-ribosomal peptide synthetase | 3586 | 3586 | 96% | RBAM_RS01840 *srfAB* surfactin non-ribosomal peptide synthetase SrfAB |
| EBA29_RS01670 *srfAC* surfactin non-ribosomal peptide synthetase | 1278 | 1278 | 93% | RBAM_RS01845 *srfAC* surfactin non-ribosomal peptide synthetase SrfAC |
| EBA29_RS01675 *srfAD* surfactin biosynthesis thioesterase SrfAD | 243 | 243 | 98% | RBAM_RS01850 *srfAD* surfactin biosynthesis thioesterase SrfAD |
| EBA29_RS01685 4'-phosphopantetheinyl transferase | 224 | 224 | 99% | RBAM_RS01875 4'-phosphopantetheinyl transferase |
| EBA29_RS03260 *purE* 5-(carboxyamino)imidazole ribonucleotide | 162 | 162 | 99% | RBAM_RS03455 *purE* 5-(carboxyamino)imidazole ribonucleotide mutase |
| EBA29_RS03265 *purK* 5-(carboxyamino)imidazole ribonucleotide | 393 | 393 | 98% | RBAM_RS03460 *purK* 5-(carboxyamino)imidazole ribonucleotide synthase |
| EBA29_RS03270 *purB* adenylosuccinate lyase | 430 | 430 | 99% | RBAM_RS03465 *purB* adenylosuccinate lyase |
| EBA29_RS03275 *purC* | 239 | 239 | 98% | RBAM_RS03470 *purC* |
| EBA29_RS03280 *purS* phosphoribosylformylglycinamidine synthase | 84 | 84 | 100% | RBAM_RS03475 *purS* phosphoribosylformylglycinamidine synthase |
| EBA29_RS03285 *purQ* phosphoribosylformylglycinamidine synthase | 227 | 227 | 99% | RBAM_RS03480 *purQ* phosphoribosylformylglycinamidine synthase |
| EBA29_RS03290 *purL* phosphoribosylformylglycinamidine synthase | 742 | 742 | 97% | RBAM_RS03485 *purL* phosphoribosylformylglycinamidine synthase |
| EBA29_RS03295 amidophosphoribosyltransferase | 476 | 476 | 99% | RBAM_RS03490 amidophosphoribosyltransferase |
| EBA29_RS03300 phosphoribosylformylglycinamidine | 346 | 346 | 92% | RBAM_RS03495 phosphoribosylformylglycinamidine |
| EBA29_RS03305 *purN* phosphoribosylglycinamide formyltransferase | 195 | 195 | 99% | RBAM_RS03500 *purN* phosphoribosylglycinamide formyltransferase |
| EBA29_RS03310 *purH* bifunctional | 512 | 512 | 99% | RBAM_RS03505 *purH* bifunctional |
| EBA29_RS03315 *purD* phosphoribosylamine--glycine ligase | 422 | 422 | 98% | RBAM_RS03510 *purD* phosphoribosylamine--glycine ligase |
| EBA29_RS07275 *fabD* ACP S-malonyltransferase | 768 | 768 | 94% | RBAM_RS07240 *fabD* ACP S-malonyltransferase |
| EBA29_RS07280 SDR family NAD(P)-dependent | 4088 | 4086 | 95% | RBAM_RS07245 SDR family NAD(P)-dependent oxidoreductase |
| EBA29_RS07285 SDR family NAD(P)-dependent | 1590 | 1590 | 94% | RBAM_RS07250 SDR family NAD(P)-dependent oxidoreductase |
| EBA29_RS07290 SDR family NAD(P)-dependent | 2903 | 2902 | 94% | RBAM_RS07255 SDR family NAD(P)-dependent oxidoreductase |
| EBA29_RS07295 SDR family NAD(P)-dependent | 2334 | 2334 | 94% | RBAM_RS07260 SDR family NAD(P)-dependent oxidoreductase |
| EBA29_RS07300 SDR family NAD(P)-dependent | 1904 | 1903 | 97% | RBAM_RS07265 SDR family NAD(P)-dependent oxidoreductase |
| EBA29_RS07305 SDR family NAD(P)-dependent | 2460 | 2459 | 94% | RBAM_RS07270 SDR family NAD(P)-dependent oxidoreductase |
| EBA29_RS07310 alpha/beta fold hydrolase | 1283 | 1283 | 93% | RBAM_RS07275 alpha/beta fold hydrolase |
| EBA29_RS07315 serine hydrolase | 363 | 363 | 94% | RBAM_RS07280 serine hydrolase |
| EBA29_RS08600 MBL fold metallo-hydrolase | 225 | 225 | 99% | RBAM_RS08525 MBL fold metallo-hydrolase |
| EBA29_RS08605 *fabD* ACP S-malonyltransferase | 289 | 289 | 94% | RBAM_RS08530 *fabD* ACP S-malonyltransferase |
| EBA29_RS08610 acyltransferase domain-containing | 324 | 324 | 99% | RBAM_RS08535 acyltransferase domain-containing protein |
| EBA29_RS08615 *fabD* ACP S-malonyltransferase | 746 | 746 | 98% | RBAM_RS08540 *fabD* ACP S-malonyltransferase |
| EBA29_RS08620 acyl carrier protein | 82 | 82 | 97% | RBAM_RS08545 acyl carrier protein |
| EBA29_RS08625 hydroxymethylglutaryl-CoA synthase | 420 | 420 | 98% | RBAM_RS08550 hydroxymethylglutaryl-CoA synthase family |
| EBA29_RS08630 enoyl-CoA hydratase/isomerase | 257 | 257 | 93% | RBAM_RS08555 enoyl-CoA hydratase/isomerase |
| EBA29_RS08635 enoyl-CoA hydratase | 249 | 249 | 100% | RBAM_RS08560 enoyl-CoA hydratase |
| EBA29_RS08640 amino acid adenylation | 4983 | 4982 | 96% | RBAM_RS08565 non-ribosomal peptide synthetase |
| EBA29_RS08645 SDR family NAD(P)-dependent | 4475 | 4475 | 93% | RBAM_RS08570 SDR family NAD(P)-dependent oxidoreductase |
| EBA29_RS08650 SDR family NAD(P)-dependent | 3511 | 3511 | 94% | RBAM_RS08575 SDR family NAD(P)-dependent oxidoreductase |
| EBA29_RS08655 amino acid adenylation | 5434 | 5433 | 95% | RBAM_RS08580 non-ribosomal peptide synthetase |
| EBA29_RS08660 methyltransferase | 2485 | 2482 | 95% | RBAM_RS08585 methyltransferase |
| EBA29_RS08665 cytochrome P450 | 403 | 403 | 96% | RBAM_RS08590 cytochrome P450 |
| EBA29_RS09255 non-ribosomal peptide synthetase | 2619 | 2619 | 98% | RBAM_RS09145 non-ribosomal peptide synthetase |
| EBA29_RS09260 amino acid adenylation | 5363 | 5363 | 96% | RBAM_RS09150 non-ribosomal peptide synthetase |
| EBA29_RS09265 hybrid non-ribosomal peptide | 3982 | 3982 | 97% | RBAM_RS09155 non-ribosomal peptide synthetase |
| EBA29_RS09270 *fabD* ACP S-malonyltransferase | 400 | 400 | 98% | RBAM_RS09160 *fabD* ACP S-malonyltransferase |
| EBA29_RS09385 amino acid adenylation | 1267 | 1267 | 96% | RBAM_RS09275 non-ribosomal peptide synthase |
| EBA29_RS09390 amino acid adenylation | 3591 | 3591 | 97% | RBAM_RS09280 non-ribosomal peptide synthetase |
| EBA29_RS09395 amino acid adenylation | 2549 | 2549 | 95% | RBAM_RS09285 non-ribosomal peptide synthetase |
| EBA29_RS09400 amino acid adenylation | 2565 | 2565 | 95% | RBAM_RS09290 non-ribosomal peptide synthetase |
| EBA29_RS09405 amino acid adenylation | 2552 | 2552 | 96% | RBAM_RS09295 non-ribosomal peptide synthetase |
| EBA29_RS11285 enoyl-CoA hydratase | 248 | 248 | 98% | RBAM_RS11085 enoyl-CoA hydratase |
| EBA29_RS11290 hydroxymethylglutaryl-CoA synthase | 415 | 415 | 99% | RBAM_RS11090 hydroxymethylglutaryl-CoA synthase family |
| EBA29_RS11295 cytochrome P450 | 384 | 384 | 98% | RBAM_RS11095 cytochrome P450 |
| EBA29_RS11300 zinc-binding dehydrogenase | 2071 | 2071 | 97% | RBAM_RS11100 zinc-binding dehydrogenase |
| EBA29_RS11305 SDR family NAD(P)-dependent | 2050 | 2050 | 96% | RBAM_RS11105 SDR family NAD(P)-dependent oxidoreductase |
| EBA29_RS11310 SDR family NAD(P)-dependent | 2572 | 2572 | 93% | RBAM_RS11110 SDR family NAD(P)-dependent oxidoreductase |
| EBA29_RS11315 SDR family NAD(P)-dependent | 5203 | 5204 | 95% | RBAM_RS11115 SDR family NAD(P)-dependent oxidoreductase |
| EBA29_RS11320 SDR family NAD(P)-dependent | 1908 | 1908 | 94% | RBAM_RS11120 SDR family NAD(P)-dependent oxidoreductase |
| EBA29_RS11325 KR domain-containing protein | 2098 | 2098 | 96% | RBAM_RS11125 KR domain-containing protein |
| EBA29_RS11330 SDR family NAD(P)-dependent | 4197 | 4196 | 94% | RBAM_RS11130 SDR family NAD(P)-dependent oxidoreductase |
| EBA29_RS11335 SDR family oxidoreductase | 245 | 245 | 88% | RBAM_RS11135 SDR family oxidoreductase |
| EBA29_RS11340 long-chain fatty acid--CoA ligase | 454 | 454 | 98% | RBAM_RS11140 long-chain fatty acid--CoA ligase |
| EBA29_RS11345 acyl carrier protein | 90 | 90 | 84% | RBAM_RS11145 acyl carrier protein |
| EBA29_RS11350 D-fructose-6-phosphate | 326 | 326 | 93% | RBAM_RS11150 D-fructose-6-phosphate amidotransferase |
| EBA29_RS11355 *fabD* ACP S-malonyltransferase | 752 | 752 | 95% | RBAM_RS11155 *fabD* ACP S-malonyltransferase |
| EBA29_RS15015 non-ribosomal peptide synthetase | 2375 | 2375 | 96% | RBAM_RS14695 non-ribosomal peptide synthetase |
| EBA29_RS15020 isochorismatase | 308 | 308 | 98% | RBAM_RS14700 isochorismatase |
| EBA29_RS15025 (2,3-dihydroxybenzoyl) adenylate | 541 | 541 | 99% | RBAM_RS14705 (2,3-dihydroxybenzoyl) adenylate synthase |
| EBA29_RS15030 *dhbC* isochorismate synthase DhbC | 398 | 398 | 96% | RBAM_RS14710 *dhbC* isochorismate synthase DhbC |
| EBA29_RS15035 2,3-dihydro-2,3-dihydroxybenzoate | 261 | 261 | 89% | RBAM_RS14715 2,3-dihydro-2,3-dihydroxybenzoate |
| EBA29_RS15105 hypothetical protein | 73 | 73 | 100% | RBAM_RS14780 hypothetical protein |
| EBA29_RS15110 hypothetical protein | 175 | 175 | 89% | RBAM_RS14785 hypothetical protein |
| EBA29_RS15115 ABC transporter ATP-binding protein | 234 | 234 | 98% | RBAM_RS14790 ABC transporter ATP-binding protein |
| EBA29_RS15120 hypothetical protein | 551 | 551 | 85% | RBAM_RS14795 hypothetical protein |
| EBA29_RS15125 circular bacteriocin, circularin | 111 | 111 | 51% | RBAM_RS14800 circular bacteriocin, circularin |
| EBA29_RS15130 hypothetical protein | 190 | 190 | 68% | RBAM_RS14805 hypothetical protein |
| EBA29_RS18005 MFS transporter | 393 | 393 | 93% | RBAM_RS17640 MFS transporter |
| EBA29_RS18010 ATP-grasp domain-containing protein | 472 | 472 | 97% | RBAM_RS17645 ATP-grasp domain-containing protein |
| EBA29_RS18015 *bacC* dihydroanticapsin 7-dehydrogenase | 253 | 253 | 99% | RBAM_RS17650 *bacC* dihydroanticapsin 7-dehydrogenase |
| EBA29_RS18020 cupin domain-containing protein | 236 | 236 | 100% | RBAM_RS17655 cupin domain-containing protein |
| EBA29_RS18025 bacilysin biosynthesis protein BacA | 204 | 204 | 99% | RBAM_RS17660 bacilysin biosynthesis protein BacA |

**Table S2** Genes involved in bacterium sporulation. Gene Bank Accession Number a brief description of their function, length (bp) and genome localization are shown

| **Accesion** | **Description** | **Gene** | **Biological function** | **bp** | **Start** | **End** |
| --- | --- | --- | --- | --- | --- | --- |
| QAR56236.1 | AbrB family transcriptional regulator protein | *abrB* | Biofilm formation | 234 | 1152487 | 1152720 |
| QAR58444.1 | amidophosphoribosyl transferase ComFC | *comFC* | Competence | 678 | 3442747 | 3443424 |
| QAR58144.1 | antimicrobial peptide SdpC family protein | *sdpC* | Killing of non-sporulating sister cells | 591 | 3131538 | 3132128 |
| QAR57299.1 | anti-sigma F factor | *spoIIAB* | Control of SigF activity; phosphorylation and inactivation of SpoIIAA | 441 | 2278932 | 2279372 |
| QAR57300.1 | anti-sigma F factor antagonist | *spoIIAA* | Control of SigF activity | 354 | 2279369 | 2279722 |
| QAR58425.1 | cell division ATP-binding protein FtsE | *ftsE* | Control of cell wall synthesis | 687 | 3426628 | 3427314 |
| QAR56620.1 | cell division septum initiation DivIVA | *divIVA* | Cell division initiation protein (septum placement) | 495 | 1540183 | 1540677 |
| QAR58619.1 | chromosome-anchoring protein RacA | *racA* | Cell division protein | 564 | 3596591 | 3597154 |
| QAR57536.1- QAR57538.1 | ComE operon competence | *comE* (*CBA*) | Genetic competence | 3537 | 2539748 | 2540385 |
| QAR57539.1 | ComE operon late competence protein ComER | *comER* | Control of biofilm formation and sporulation via the phosphorelay | 822 | 2541058 | 2541879 |
| QAR57447.1- QAR57539.1 | ComG operon competence | *comG* (*GFEDCBA*) | Genetic competence, DNA uptake | 3918 | 2461403 | 2463875 |
| QAR56177.1 | ComG operon repressor ComZ | *comZ* | Genetic competence | 192 | 1101724 | 1101915 |
| QAR56775.1 | competence damage-inducible protein A | *cinA* | competence-damage inducible regulator | 1251 | 1694685 | 1695935 |
| QAR58051.1 | competence pheromone ComX-like protein | *comX* | Triggers the production of surfactin Quorum sensing | 165 | 3038106 | 3038270 |
| QAR58445.1 | competence protein ComFB | *comFB* | Competence | 285 | 3443442 | 3443726 |
| QAR58052.1 | competence regulatory protein ComQ | *comQ* | Regulation of quorum sensing | 912 | 3038270 | 3039181 |
| QAR56103.1 | competence transcription factor ComK | *comK* | Regulation of genetic competence and DNA uptake | 579 | 1032477 | 1033055 |
| QAR56200.1 | competence-induced CoiA-like protein | *coiA* | Genetic competence | 1161 | 1123828 | 1124988 |
| QAR56763.1 | DNA translocase FtsK/SpoIIIE | *spoIIIE* | Chromosome partition during sporulation | 2367 | 1680106 | 1682466 |
| QAR58446.1 | DNA/RNA helicase | *comFA* | Competence | 1386 | 3443784 | 3445169 |
| QAR56699.1 | GTP-sensing transcriptional pleiotropic repressor CodY | *codY* | Swarming motility | 780 | 1618282 | 1619061 |
| QAR55478.1 | kinase A inhibitor protein | *kipI* | Control of the phosphorelay, initiation of sporulation | 732 | 403954 | 404685 |
| QAR55479.1 | KipI antagonist protein | *kipA* | Control of the phosphorelay, initiation of sporulation | 1014 | 404688 | 405695 |
| QAR56666.1 | L-serine deaminase subunit alpha | *sdaAA* | Serine utilization | 903 | 1586889 | 1587791 |
| QAR56665.1 | L-serine deaminase subunit beta | *sdaAB* | Serine utilization | 663 | 1586206 | 1586868 |
| QAR56300.1 | phosphatase RapA inhibitor protein | *phrA* | Control of sporulation initiation | 135 |  |  |
| QAR55447.1 | phosphatase RapC inhibitor protein PhrC | *phrC* | Control of ComA activity | 120 | 376713 | 376832 |
| QAR57690.1 | prepilin peptidase | *comC* | Genetic competence | 753 | 2682459 | 2683211 |
| QAR56299.1 | response regulator aspartate phosphatase A 1 | *rapA1* | Control of sporulation initiation | 1131 | 1208385 | 1209521 |
| QAR57104.1 | response regulator aspartate phosphatase A 2 | *rapA2* | Control of sporulation initiation | 1137 | 2110371 | 2111507 |
| QAR55446.1 | response regulator aspartate phosphatase C | *rapC* | Control of ComA activity | 1149 | 375581 | 376729 |
| QAR58129.1 | response regulator aspartate phosphatase H | *rapH* | Control of sporulation initiation and ComA activity | 1131 | 3115660 | 3116790 |
| QAR58708.1-QAR58714.1 | spore coat polysaccharide biosynthesis protein Sps | *spsGFEDCBA* | Spore coat polysaccharide synthesis | 7098 | 3683966 | 3689307 |
| QAR56786.1 | spore coat protein CotE | *cotE* | Assembly of the outer spore coat | 546 | 1706519 | 1707064 |
| QAR55481.1 | spore germination lipase LipC | *lipC* | Spore germination | 642 | 406527 | 407168 |
| QAR58146.1 | sporulation delaying SdpA family protein | *sdpA* | Maturation of the SdpC toxin | 531 | 3133062 | 3133592 |
| QAR58145.1 | sporulation delaying SdpB family protein | *sdpB* | Maturation of the SdpC toxin | 945 | 3132121 | 3133065 |
| QAR56467.1 | sporulation histidine kinase A | *kinA* | Sporulation-specific ATP-dependent protein histidine kinase | 1821 | 1353420 | 1355240 |
| QAR58025.1 | sporulation histidine kinase B | *kinB* | Initiation of sporulation | 1284 | 3014006 | 3015289 |
| QAR56515.1 | sporulation histidine kinase C | *kinC* | Two-component sensor histidine kinase | 1305 | 1394757 | 1396061 |
| QAR56430.1 | sporulation histidine kinase D | *kinD* | Histidine kinase phosphorylating Spo0A | 1518 | 1316668 | 1318185 |
| QAR56416.1 | sporulation histidine kinase E | *kinE* | Initiation of sporulation | 2220 | 1305013 | 1307232 |
| QAR57549.1 | sporulation inhibitor A | *sda* | Developmental checkpoint protein, controls the phosphorylation status of Spo0A | 141 | 2547311 | 2547451 |
| QAR56882.1 | sporulation inhibitor of replication protein SirA | *sirA* | Control of chromosome copy number | 447 | 1852447 | 1852893 |
| QAR57676.1 | sporulation initiation phosphotransferase B | *spo0B* | Initiation of sporulation | 579 | 2672059 | 2672637 |
| QAR58630.1 | sporulation initiation phosphotransferase F | *spo0F* | Initiation of sporulation | 375 | 3607283 | 3607657 |
| QAR56609.1 | sporulation sigma factor SigE | *sigE* | Transcription of sporulation genes (early mother cell) | 720 | 1532484 | 1533203 |
| QAR57298.1 | sporulation sigma factor SigF | *sigF* | Transcription of sporulation genes (early forespore) | 768 | 2278153 | 2278920 |
| QAR56610.1 | sporulation sigma factor SigG | *sigG* | Transcription of sporulation genes (late forespore) | 783 | 1533344 | 1534126 |
| QAR57556.1 | sporulation sigma factor SigK | *sigK* | Late mother cell-specific gene expression | 726 | 2552118 | 2552843 |
| QAR57401.1 | stage 0 sporulation protein A | *spo0A* | Biofilm formation | 801 | 2423531 | 2424331 |
| QAR58589.1 | stage II sporulation protein D | *spoIID* | Dissolution of the septal cell wall | 1032 | 3574630 | 3575661 |
| QAR55159.1 | stage II sporulation protein E | *spoIIE* | Control of SigF activity, formation of the asymmetric septum | 2499 | 71116 | 73605 |
| QAR57307.1 | stage II sporulation protein M | *spoIIM* | Dissolution of the septal cell wall | 645 | 2285109 | 2285753 |
| QAR58567.1 | stage II sporulation protein Q | *spoIIQ* | Forespore encasement by the spore coat | 870 | 3558323 | 3559192 |
| QAR58613.1 | stage II sporulation protein R | *spoIIR* | Control of SigE activation | 681 | 3592502 | 3593182 |
| QAR57423.1 | stage III sporulation protein AA | *spoIIIAA* | Activation of SigG | 924 | 2442158 | 2443081 |
| QAR57422.1 | stage III sporulation protein AB | *spoIIIAB* | Activation of SigG | 516 | 2441649 | 2442164 |
| QAR57421.1 | stage III sporulation protein AC | *spoIIIAC* | Activation of SigG | 207 | 2441420 | 2441626 |
| QAR57420.1 | stage III sporulation protein AD | *spoIIIAD* | Activation of SigG | 450 | 2441017 | 2441466 |
| QAR57419.1 | stage III sporulation protein AE | *spoIIIAE* | Activation of SigG | 1209 | 2439776 | 2440984 |
| QAR57418.1 | stage III sporulation protein AF | *spoIIIAF* | Activation of SigG | 621 | 2439139 | 2439759 |
| QAR57417.1 | stage III sporulation protein AG | *spoIIIAG* | Activation of SigG | 693 | 2438454 | 2439146 |
| QAR57416.1- | stage III sporulation protein AH- | *spoIIIAH* | Activation of SigG, forespore encasement by the spore coat | 651 | 2437800 | 2438450 |
| QAR57238.1 | stage IV sporulation protein A | *spoIVA* | Spore cortex formation and coat assembly | 1479 | 2224309 | 2225787 |
| QAR56597.1 | stage V sporulation protein D | *spoVD* | Spore morphogenesis | 1920 | 1513418 | 1515337 |
| QAR56601.1 | stage V sporulation protein E | *spoVE* | Spore cortex peptidoglycan synthesis | 1101 | 1519502 | 1520602 |
| QAR57696.1 | stage VI sporulation protein D | *spoVID* | Spore coat assembly | 1887 | 2689059 | 2690939 |
| QAR58049.1 | two-component response regulator protein ComA | *comA* | Regulation of genetic competence and quorum sensing | 645 | 3035078 | 3035722 |
| QAR58050.1 | two-component sensor histidine kinase protein ComP | *comP* | Regulation of genetic competence and quorum sensing | 2292 | 3035803 | 3038094 |

**Table S3** Genes involved in plant-bacterium interactions. Gene Bank Accession Number a brief description of their function, length (bp) and genome localization are shown

| **Accesion** | **Description** | **Gene** | **Biological function** | **bp** | **Start** | **End** |
| --- | --- | --- | --- | --- | --- | --- |
| QAR58182.1 | iron-enterobactin transporter ATP-binding protein | *yusV* | Acquisition of iron | 825 | 3162216 | 3163040 |
| QAR55252.1 | KinB-signaling pathway activation protein | *kbaA* | Activation of the KinB-dependent pathway to sporulation, control of the phosphorelay | 597 | 154005 | 154601 |
| QAR57012.1 | FMN-dependent NADH-azoreductase 1 | *azoR1* | Azoreductase | 627 | 2037963 | 2038589 |
| QAR58255.1 | FMN-dependent NADH-azoreductase 2 | *azoR2* | Azoreductase | 636 | 3231925 | 3232560 |
| QAR57253.1 | bacillithiol biosynthesis thiol disulfide oxidoreductase protein | *ypdA* | Bacillithiol biosynthesis | 984 | 2237941 | 2238924 |
| QAR57779.1 | two-component sensor histidine kinase protein LytS | *lytS* | Biofilm | 1782 | 2772766 | 2774547 |
| QAR55194.1 | RNA polymerase sigma-H factor | *sigH* | Biofilm formation | 657 | 111329 | 111985 |
| QAR55273.1 | RNA polymerase sigma factor SigW | *sigW* | Biofilm formation | 564 | 182480 | 183043 |
| QAR55328.1 | two-component sensor histidine kinase protein GlnK | *glnK* | Biofilm formation | 1293 | 236779 | 238071 |
| QAR57267.1 | two-component sensor histidine kinase protein ResE | *resE* | Biofilm formation | 1782 | 2251647 | 2253428 |
| QAR57439.1 | anti-repressor SinI | *sinI* | Biofilm formation | 174 | 2457494 | 2457667 |
| QAR57440.1 | repressor SinR | *sinR* | Biofilm formation | 342 | 2457701 | 2458036 |
| QAR58990.1 | Levansucrase | *sacB* | Biofilm formation | 1422 | 3949219 | 3950640 |
| QAR58335.1 | transcriptional regulator protein | *slrR* | Biofilm formation and autolysis | 456 | 3309553 | 3310008 |
| QAR57986.1 | YuaB family protein (bslA) | *bslA* | Biofilm formation, control of entry into sporulation via the phosphorelay | 543 | 2976466 | 2977002 |
| QAR56236.1 | AbrB family transcriptional regulator protein | *abrB* | Biofilm formation, sporulation | 234 | 1152487 | 1152720 |
| QAR57401.1 | stage 0 sporulation protein A | *spo0A* | Biofilm formation, sporulation | 801 | 2423531 | 2424331 |
| QAR56263.1 | galactose-1-phosphate uridylyltransferase 1 | *galT1* | Cellobiose degradation | 1500 | 1174600 | 1176099 |
| QAR56264.1 | UDP-glucose 4-epimerase 1 | *galE1* | Cellobiose degradation | 993 | 1176101 | 1177093 |
| QAR56265.1 | galactokinase 1 | *galK1* | Cellobiose degradation | 1170 | 1177097 | 1178266 |
| QAR56266.1 | PTS system lactose-specific EIICB component | *lacE* | Cellobiose degradation | 1698 | 1178282 | 1179979 |
| QAR56267.1 | PTS system lactose-specific phosphotransferase enzyme IIA component | *lacF* | Cellobiose degradation | 315 | 1179991 | 1180305 |
| QAR56647.1 | YlzA family protein | *ylzA* | Conserved hypothetical protein (rmaA) Control of biofilm formation | 270 | 1569646 | 1569915 |
| QAR56578.1 | cell fate regulator protein YlbF | *ylbF* | Control for community development | 450 | 1497646 | 1498095 |
| QAR56785.1 | YmcA family protein | *ymcA* | Control of community development | 432 | 1705835 | 1706266 |
| QAR58745.1 | repressor/antirepressor protein | *slrA* | Control of motility and biofilm formation | 162 | 3718260 | 3718421 |
| QAR55209.1 | elongation factor Tu | *tuf* | Elicitation of plant basal defence | 1191 | 127582 | 128772 |
| QAR58434.1 | flagellar hook-associated protein FliD | *fliD* | Elicitation of plant defence | 1521 | 3434709 | 3436229 |
| QAR58435.1 | flagellin protein | *hag* | Elicitation of plant defence | 996 | 3436481 | 3437476 |
| QAR58440.1 | flagellar hook-associated protein FlgK | *flgK* | Elicitation of plant defence | 1503 | 3439888 | 3441405 |
| QAR58452.1-QAR58460.1 | tuaH- tuaA | *tuaH- tuaA- tagO* | Elicitation of plant defence  Teichuronic acid biosynthesis | 9849 | 3451407 | 3459793 |
| QAR57441.1- QAR57443.1 | yqxM- sipW- tasA operon | *yqxM-sipW-tasA* | Essencial for biofilm formation | 2043 | 2458869 | 2459490 |
| QAR56774.1 | CDP-diacylglycerol--glycerol-3-phosphate 3-phosphatidyltransferase | *pgsA* | Essencial for polyglutamic synthesis  Biosynthesis of phospholipids | 582 | 1694086 | 1694667 |
| QAR56730.1 | swarming motility protein SwrB | *swrB* | Essencial for swarming motility | 483 | 1645282 | 1645755 |
| QAR57425.1 | elongation factor P | *efp* | Essencial for swarming motility | 558 | 2443587 | 2444144 |
| QAR58421.1 | swarming motility protein SwrAA | *swrAA* | Essencial for swarming motility | 354 | 3421998 | 3422351 |
| QAR56838.1 | Na+/galactoside symporter protein | *xynP* | Extracellular degradation of cell walls | 1392 | 1812006 | 1813397 |
| QAR56839.1 | beta-xylosidase | *xynB* | Extracellular degradation of cell walls | 1602 | 1813440 | 1815041 |
| QAR56927.1 | glucuronoarabinoxylan endo-1,4-beta-xylanase | *xynC* | Extracellular degradation of cell walls | 1272 | 1887773 | 1889044 |
| QAR56928.1 | non-reducing end alpha-L-arabinofuranosidase | *xynD* | Extracellular degradation of cell walls | 1536 | 1889106 | 1890644 |
| QAR58575.1 | endo-1,4-beta-xylanase | *xynA* | Extracellular degradation of plant cell walls | 642 | 3564153 | 3564794 |
| QAR56262.1 | arabinogalactan endo-1,4-beta-galactosidase protein | *ganA* | Extracelular degradation of plant cell walls | 1113 | 1173436 | 1174548 |
| QAR55401.1 | 6-phospho-beta-glucosidase | *bglC* | Glucan degradation | 1437 | 315878 | 317314 |
| QAR56083.1 | M42 aminopeptidase protein | *yhfE* | Glucan degradation | 1032 | 1012676 | 1013716 |
| QAR58829.1 | beta-glucanase | *bglS* | Glucan degradation | 732 | 3792818 | 3793549 |
| QAR56430.1 | sporulation histidine kinase D | *kinD* | Histidine kinase phosphorylating Spo0A | 1518 | 1316668 | 1318185 |
| QAR57109.1 | oxygen two-component sensor histidine kinase protein NreB | *nreB* | Histidine kinase protein | 1074 | 2115989 | 2117062 |
| QAR56268.1 | 6-phospho-beta-galactosidase | *lacG* | Hydrolyzation of phospholactose | 1401 | 1180369 | 1181769 |
| QAR58025.1 | sporulation histidine kinase B | *kinB* | Initiation of sporulation | 1284 | 3014006 | 3015289 |
| QAR56269.1 | lactose phosphotransferase system repressor | *lacR* | Lactose metabolism | 762 | 1182022 | 1182783 |
| QAR56433.1 | flagellar motor protein MotB | *motB* | Motility and chemotaxis | 786 | 1318866 | 1319651 |
| QAR56434.1 | flagellar motor protein MotA | *motA* | Motility and chemotaxis | 816 | 1319623 | 1320438 |
| QAR58320.1- QAR58334.1 | Operon for capsular poly-saccharide biosynthesis | *epsO*-*epsA* | Necessary for biofilm formation | 15471 | 3294558 | 3308601 |
| QAR55416.1 | 4'-phosphopantetheinyl transferase protein | *sfp* | Necessary for surfactin synthesis | 675 | 348543 | 349217 |
| QAR55417.1 | membrane protein | *yczE* | Necessary for surfactin synthesis | 648 | 349316 | 349963 |
| QAR57086.1 | 3-phytase | *phy* | Phosphate availability | 1152 | 2096262 | 2097413 |
| QAR55519.1 | EcsC family protein | *ydbA* | Protein secretion to extracelular matrix | 804 | 444196 | 444999 |
| QAR56068.1- QAR56070.1 | ABC transporter ATP-binding protein | *ecsABC* | Protein secretion to extracelular matrix |  | 996851 | 998113 |
| QAR58049.1 | two-component response regulator protein ComA | *comA* | Regulation of genetic competence and quorum sensing | 645 | 3035078 | 3035722 |
| QAR58050.1 | two-component sensor histidine kinase protein ComP | *comP* | Regulation of genetic competence and quorum sensing | 2292 | 3035803 | 3038094 |
| QAR58052.1 | competence regulatory protein ComQ | *comQ* | Regulation of quorum sensing | 912 | 3038270 | 3039181 |
| QAR55749.1 | swarming motility protein SwrC | *swrC* | Selfresistance for surfactin | 3144 | 671014 | 674157 |
| QAR56467.1 | sporulation histidine kinase A | *kinA* | Sporulation-specific ATP-dependent protein histidine kinase | 1821 | 1353420 | 1355240 |
| QAR58683.1 | phosphate acetyltransferase | *pta* | Strongly upregulated by root exudate | 972 | 3657060 | 3658031 |
| QAR55410.1- QAR55413.1 | Surfactin operon | *srfAABCD* | Surfactin, surface motility and biofilm formation | 26085 | 331671 | 346344 |
| QAR56699.1 | GTP-sensing transcriptional pleiotropic repressor CodY | *codY* | Swarming motility | 780 | 1618282 | 1619061 |
| QAR56700.1- QAR56728.1 | Operon for flagellar synthesis and chemotaxis | *flgB*-*che*D | Swarming motility | 22752 | 1619803 | 1643963 |
| QAR56729.1 | RNA polymerase sigma factor SigD | *sigD* | Swarming motility | 765 | 1644490 | 1645254 |
| QAR58448.1- QAR58449.1 | Operon of two-component regulatory system DegSU | *degUS* | swarming motility, biofilm formation, complex colony architecture, and protease production | 1854 | 3446989 | 3446912 |
| QAR58504.1 | alpha-acetolactate decarboxylase | *alsD* | Synthesis of butanodiol/acetoin | 768 | 3506901 | 3507668 |
| QAR58505.1 | acetolactate synthase | *alsS* | Synthesis of butanodiol/acetoin | 1713 | 3507729 | 3509441 |
| QAR58506.1 | LysR family transcriptional regulator protein | *alsR* | Synthesis of butanodiol/acetoin | 909 | 3509598 | 3510506 |
| QAR58053.1 | degradation enzyme regulation protein DegQ | *degQ* | Synthesis of exoenzymes and of extracellular poly-gamma-glutamate | 141 | 3039333 | 3039473 |
| QAR58488.1- QAR58490.1 | poly-gamma-glutamate biosynthesis protein CapACB | *capACB* | Synthesis of PGA | 2781 | 3493734 | 3494217 |
| QAR55453.1 | iron ABC transporter substrate-binding protein | *yclQ* | Transport/Binding protein and lipoproteins | 945 | 381616 | 382560 |
| QAR58051.1 | competence pheromone ComX-like protein | *comX* | Triggers the production of surfactin Quorum sensing | 165 | 3038106 | 3038270 |
| QAR55983.1 | nitrilase family protein | *yhcX* | Trp-dependent IAA synthesis | 1539 | 918451 | 919989 |
| QAR57022.1 | aldehyde dehydrogenase (NAD(+)) | *dhaS* | Trp-dependent IAA synthesis | 1488 | 2042332 | 2043819 |
| QAR58734.1 | GNAT family N-acetyltransferase protein | *ysnE* | Trp-dependent IAA synthesis | 459 | 3708306 | 3708764 |
| QAR56515.1 | sporulation histidine kinase C | *kinC* | Two-component sensor histidine kinase | 1305 | 1394757 | 1396061 |
